# Apathy in small vessel cerebrovascular disease is associated with deficits in effort-based decision making

**DOI:** 10.1093/brain/awab013

**Published:** 2021-03-18

**Authors:** Youssuf Saleh, Campbell Le Heron, Pierre Petitet, Michele Veldsman, Daniel Drew, Olivia Plant, Ursula Schulz, Arjune Sen, Peter M Rothwell, Sanjay Manohar, Masud Husain

**Affiliations:** 1 Nuffield Department of Clinical Neurosciences, University of Oxford, Level 6, West Wing, John Radcliffe Hospital, Oxford, OX3 9DU, UK; 2 Department of Experimental Psychology, University of Oxford, Anna Watts Building, Radcliffe Observatory Quarter, Oxford, OX2 6GG, UK; 3 New Zealand Brain Research Institute, Christchurch 8011, New Zealand; 4 Department of Medicine, University of Otago, Christchurch 8011, New Zealand; 5 Oxford Epilepsy Research Group, NIHR Biomedical Research Centre, Nuffield Department of Clinical Neurosciences, John Radcliffe Hospital, Oxford, OX3 9DU, UK; 6 Wolfson Centre for Prevention of Stroke and Dementia, Nuffield Dept Clinical Neurosciences, University of Oxford, UK; 7 NIHR Oxford Biomedical Research Centre, Oxford, UK; 8 Wellcome Centre for Integrative Neuroimaging, University of Oxford, UK

**Keywords:** apathy, cerebrovascular small vessel disease, drift diffusion model, decision-making, reward

## Abstract

Patients with small vessel cerebrovascular disease frequently suffer from apathy, a debilitating neuropsychiatric syndrome, the underlying mechanisms of which remain to be established. Here we investigated the hypothesis that apathy is associated with disrupted decision making in effort-based decision making, and that these alterations are associated with abnormalities in the white matter network connecting brain regions that underpin such decisions. Eighty-two patients with MRI evidence of small vessel disease were assessed using a behavioural paradigm as well as diffusion weighted MRI. The decision-making task involved accepting or rejecting monetary rewards in return for performing different levels of physical effort (hand grip force). Choice data and reaction times were integrated into a drift diffusion model that framed decisions to accept or reject offers as stochastic processes approaching a decision boundary with a particular drift rate. Tract-based spatial statistics were used to assess the relationship between white matter tract integrity and apathy, while accounting for depression. Overall, patients with apathy accepted significantly fewer offers on this decision-making task. Notably, while apathetic patients were less responsive to low rewards, they were also significantly averse to investing in high effort. Significant reductions in white matter integrity were observed to be specifically related to apathy, but not to depression. These included pathways connecting brain regions previously implicated in effort-based decision making in healthy people. The drift rate to decision parameter was significantly associated with both apathy and altered white matter tracts, suggesting that both brain and behavioural changes in apathy are associated with this single parameter. On the other hand, depression was associated with an increase in the decision boundary, consistent with an increase in the amount of evidence required prior to making a decision. These findings demonstrate altered effort-based decision making for reward in apathy, and also highlight dissociable mechanisms underlying apathy and depression in small vessel disease. They provide clear potential brain and behavioural targets for future therapeutic interventions, as well as modelling parameters that can be used to measure the effects of treatment at the behavioural level.

## Teaser

Apathy is common in patients with cerebrovascular small vessel disease. Saleh *et al*. show that apathy is associated with altered effort-based decision making for reward, and that these changes correlate with abnormalities in the white matter network connecting the medial frontal regions and basal ganglia

## Introduction

Small vessel cerebrovascular disease (SVD) is a common disorder which primarily affects cerebral microvessels.^1^ Initially thought to be innocuous, SVD is currently considered to be the largest contributor to vascular dementia worldwide, and is typically characterized by white matter hyperintensities on MRI.^2^^-^^4^ Radiological and pathological features also include lacunes, subcortical infarcts, microbleeds, enlarged perivascular spaces and cerebral atrophy.^1^^,^^5^ Up to 50% of patients with SVD and 92% of individuals with vascular dementia suffer from apathy,^6^^,^^7^ a syndrome associated with poor functional outcomes^8^ and lower quality of life.^9^ In patients with SVD, apathy is associated with significantly increased risk of dementia^4^^,^^5^ as well as mortality.^10^ Despite its burden on patients and their carers,^11^ there are no current licensed treatments for apathy, partly due to a poor understanding of its underlying mechanisms.^12^

Apathy has been conceptualized as a deficit in ‘motivated voluntary behaviour’—a deficit in linking motivation to the initiation of action.^13^ A novel approach to quantify motivated behaviour in patients is to examine performance on paradigms that assess effort-based decision making for rewards. These tasks, translated from studies in animal models,^14^^-^^16^ have played an important role in phenotyping apathetic behaviour in humans by probing different phases of decision making.^17^ An important component of deciding whether to take a behavioural course of action involves weighing up its perceived benefits (e.g. potential reward), against its subjective costs, (e.g. effort required to obtain that reward). By parametrically manipulating reward and effort within an experimental task, it is possible to characterize the behavioural changes that underlie apathy.^18^^,^^19^

*A priori*, at least three distinct mechanisms might potentially contribute to apathetic behaviour when deciding whether to allocate effort to obtain rewarding outcomes. First, it is possible that patients with apathy might be hypersensitive to effort, and therefore not willing to invest in behaviours that they perceive to be too physically or mentally demanding even when the reward outcome is high. Alternatively, they might be less incentivized by rewards, and accept only those few offers with the highest reward outcomes regardless of the effort requirement. Finally, they may be less inclined to accept offers overall, irrespective of the associated reward or effort. A recent investigation in Parkinson’s disease revealed that apathetic patients performing an effort-based decision making task were less willing to exert effort in return for reward, particularly when the levels of reward were low.^20^ Crucially, they were just as willing as non-apathetic patients with Parkinson’s disease to allocate high levels of effort when the rewards were high. Thus, apathy in Parkinson’s disease is associated with insensitivity to rewards for low magnitudes of reward, rather than hypersensitivity to effort. This has therapeutic implications because dopaminergic therapy, known to ameliorate apathy in some patients with Parkinson’s disease, can improve reward sensitivity in Parkinson’s disease.^21^^,^^22^

It is not clear though if the same type of deficit underlies effort-based decision making in SVD, or if apathetic behaviour across different diseases might be characterized by very different underlying impairments. While reward insensitivity characterizes apathy in Parkinson’s disease, it is possible that apathetic patients with SVD are hypersensitive to effort, insensitive to reward or generally less willing to initiate actions. Some evidence for reduced reward responsiveness to low levels of reward comes from an investigation in CADASIL (cerebral autosomal dominant arteriopathy with subcortical infarcts and leukoencephalopathy), a heritable form of SVD.^23^ However, this study examined a total of only 19 patients, and it remains an open question as to whether the findings in this rare genetic condition would translate to the much more prevalent, sporadic form of SVD that typically occurs later in life.

Another important related issue concerns clarification of the neural basis of apathy in SVD.^24^ It is evident from functional neuroimaging studies in healthy subjects that distinct brain regions are activated while performing effort-based decision-making tasks.^25^ These are typically located within frontal and striatal regions, including the medial orbitofrontal cortex [also referred to as the ventromedial prefrontal cortex (vmPFC)], anterior cingulate cortex (ACC) and basal ganglia, including in particular the ventral striatum.^26^^-^^29^ Within this network, different regions may contribute to specific components of decision making. For example, activation within medial orbitofrontal cortex and ventral striatum correlates positively with increasing reward magnitude,^28^^-^^30^ whereas lesions to these areas can significantly reduce responsiveness to reward.^31^^,^^32^ On the other hand, the ACC appears to compute the net subjective value of decisions by integrating reward and effort signals.^27^^,^^28^^,^^33^ It has long been known that lesions there can result in severe forms of apathy manifest as akinetic mutism.^34^^,^^35^ Together these findings suggest that motivated behaviour in healthy individuals might be regulated by a circumscribed fronto-striatal network, with deficits within this network potentially giving rise to clinical apathy.

Is apathy in SVD associated with disruption to a specific brain network? Several studies have investigated the association between motivational deficits and structural brain changes in SVD, largely focusing on white matter lesions and lacunar infarcts.^36^^-^^39^ While some report a positive association between these measures and the development of apathy,^36^^,^^37^ the findings have been inconsistent.^39^^,^^40^ There are several possible reasons. First, previous investigations have not consistently used the same instruments to define apathy, and a recent review highlighted the paucity of studies using validated apathy questionnaires to infer associations in neuroimaging studies.^41^ Second, recruitment of SVD patients has varied considerably across investigations, from early stage non-demented patients to those with significant cognitive decline.^36^^,^^38^ This means that patient groups in some studies have significantly greater lesion burden than others which may alter the associations of interest. In more advanced cases of SVD, lesion load is more likely to represent general disease progression rather than highlight specific neural underpinnings of neuropsychiatric syndromes.

One way to mitigate against this is by using more sensitive diffusion-weighted imaging (DWI) measures such as fractional anisotropy (FA), which can quantify microstructural integrity in normal-appearing white matter.^42^^-^^44^ These imaging indices might facilitate better detection of network deficits in patients with early stages of SVD whose overall disease burden is low. Two recent investigations have reported that apathy is associated with microstructural deficits within frontal and striatal regions,^6^^,^^45^ but neither of these studies were designed to investigate the behavioural or cognitive mechanisms underlying apathy and relate these to brain networks. Furthermore, there are concerns about how much of the effects attributed to apathy might in fact be due to depression, which is also common in SVD.^46^ While the two syndromes often coexist, apathy is increasingly recognized as a distinct entity with its own significant clinical impacts.^47^^-^^49^ It would therefore be important to demonstrate in a large sample of sporadic, late-onset SVD that brain mechanisms underlying apathy are both behaviourally and anatomically independent from depression.

Here we report on the first investigation in sporadic SVD combining performance metrics from an effort-based decision-making task with DWI. Specifically, we used Bayesian drift diffusion modelling (DDM), a well validated modelling technique,^50^^-^^52^ to relate behavioural metrics to DWI, and attempting to account for any effects of depression in both behavioural and imaging analyses. Our aims were to answer the following questions: What are the behavioural changes of apathy associated with effort-based decision making for reward in sporadic SVD? Does apathy in SVD have distinct neural underpinnings that are dissociable from depression? Is there a clear relationship between behavioural and neuroimaging changes in apathy*?*

## Materials and methods

### Ethics

This study was approved by the local ethics committee. Informed written consent was obtained from all participants and all testing was conducted in line with the declaration of Helsinki.

### Participants

We screened 104 patients with expected MRI evidence of SVD from the Oxford Vascular Study (OXVASC) and Oxford neurology clinics between January 2015 and February 2019. The OXVASC study is a population-based cohort study of patients who underwent MRI following a transient ischaemic attack (TIA) or minor ischaemic stroke.^53^^,^^54^ They were recruited to our study following neuroimaging and therefor a proportion were subsequently excluded if there was no evidence of SVD on their scans. Additionally, patients were recruited from three Oxford neurology clinics: (i) The memory clinic, investigating patients with cognitive complaints; (ii) the TIA clinic, investigating patients with possible stroke/TIA; and (iii) the first fit clinic, investigating patients with first possible seizure.

Clinic patients were recruited after confirmed SVD on MRI which included white matter hyperintensities of presumed vascular origin, as defined by the STRIVE criteria,^5^ lacunar and/or subcortical infarcts. Such a group of patients is likely to have heterogeneous underlying pathology, e.g. some might also have concomitant Alzheimer pathology. Therefore, we also deployed the following exclusion criteria: (i) known diagnosis of dementia, including clinical diagnoses of Alzheimer’s or vascular dementia; (ii) evidence or history suggestive of any concomitant neurodegenerative or neuroinflammatory disease other than SVD; (iii) participants without an established diagnosis of dementia who were unable to understand and engage with the behavioural testing protocol; (iv) physical disability preventing participants squeezing a hand-held device in response to visual stimuli; and (v) cortical strokes > 1.5 cm longest dimension on MRI (slice thickness = 3 mm).

Twenty-two participants were excluded from the initial recruitment, leaving 82 who underwent behavioural testing (Table 1). Of these, 67 had complete diffusion neuroimaging, and 62 had complete structural and DWI. Sample size was considered adequate based on previous studies using the behavioural task in patient populations, which used 19 and 39 cases of CADASIL and Parkinson’s disease, respectively.^20^^,^^23^ A study flow chart can be viewed in the Supplementary material for more details on exclusion and incomplete data. Disease severity was graded using the Fazekas scale by a clinician.^55^^,^^56^

**Table 1:** Demographics

Measure	Patient cohort *n *=* *82
Age, years	68 (11)
Gender, female/male	37/45 (45%/55%)
Apathy^a^, AES, range 0–72	32 (8)
Depression, BDI, range 0–63	10 (7)
Cognition^b^, ACE-III, range 0–100	90 (8)
Quality of life, CANTRIL, range 0–10	7.32 (1.64)
Apathetic^a^ (AES > 34), yes/no	26/54 (33%/67%)

Statistics are presented as mean (SD) unless otherwise shown.

a Data missing for two participants.

b Data missing for three participants.

### Clinical measures

Apathy was measured using the Apathy Evaluation Scale (AES), a well described instrument that has been used to characterize apathy across neurological conditions, including SVD.^20^^,^^23^^,^^57^ Here we used the self-report version (AES-S). For most of the analyses reported below, the AES score was used as a continuous variable; where we compared groups with apathy and without, we used a cut off AES score of >34 to define apathy. This cut-off score has been validated across neurological conditions.^58^^,^^59^ Depression was measured using the Beck Depression Inventory (BDI) and cognitive function using the Addenbrooke's Cognitive Examination III (ACE-III). Quality of life was recorded using the Cantril quality of life ladder. This involves a single response to a visual scale quantifying their perceived quality of life from 1 to 10 at the time of testing.^60^

### Effort-based decision-making task

Participants completed an effort-based decision-making task previously used to investigate apathy in both healthy participants^27^ and patients with neurological disease.^18^^,^^20^^,^^23^ This paradigm was designed in psychtoolbox (psychtoolbox.org) within MATLAB and administered on a mounted desktop.

**Figure 1:**
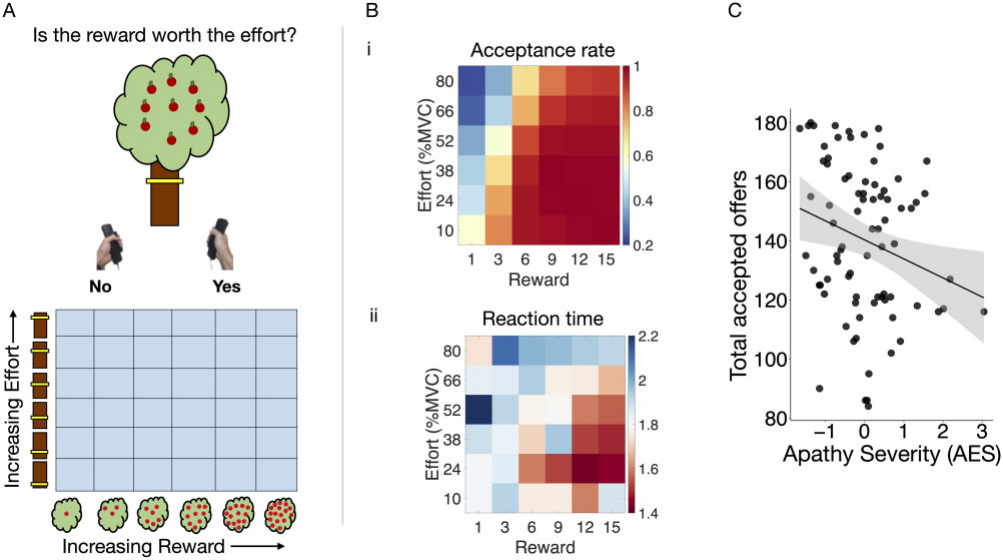
**Effort-based decision making-task and overall performance as a function of apathy.** (**A**) On a trial-by-trial basis, participants were offered monetary rewards (virtual apples) in return for physical effort (height of yellow bar). By varying the amount of reward and effort, their acceptance or rejection of different reward-effort combinations could be mapped in a two-dimensional decision space of reward and effort (6 × 6 grid, *bottom*). (**B**) Acceptance rates and reaction times in each section of the decision space. (**i**) Patients accepted more offers and reacted faster as rewards increased (heat map becomes more red from *left* to *right*). (**ii**) Inversely they rejected more offers and reacted slower as the effort increased (heat map more blue from *bottom* to *top*). (**C**) Fewer offers were accepted overall with increasing apathy severity (*z*-scored values on *x*-axis).

Participants were offered monetary rewards in return for physical effort (Fig. 1A). On a trial by trial basis, they were asked to consider if the reward was worth the effort on offer. They could either accept or reject the offer by gently squeezing the handheld device on the side of the preferred response (‘Yes/No’ shown on the screen). If an offer was accepted, participants had 5 s to squeeze in proportion to the required effort level and sustain this effort for at least 1 s. No explicit instructions related to time were given at the outset of the experiment. They were only informed that the more apples they collected, the more money they would gain in the end. Online force feedback allowed participants to visualize their proximity to the target force required. Successful effort allocation was followed by a feedback phase confirming the amount of reward secured on the trial as well as the overall reward earnings up to that point. On the other hand, a rejected offer was followed by a short pause followed by commencement to the next trial. This ensured that accepted and rejected trials were matched for trial length throughout the experiment.

Rewards were represented by the number of virtual apples on a tree (1, 3, 6, 9, 12, or 15) and effort by the height of a yellow bar on the tree trunk (Fig. 1A, top). Higher bar positions indicated a higher effort requirement to obtain the reward on offer on that trial. Effort was measured as force expressed by squeezing calibrated handheld dynamometers (SS25LA, BIOPAC Systems). Crucially, the force required was a percentage of each participant’s own maximum voluntary contraction (10, 24, 38, 52, 66 or 80%) established as a mean of three trials prior to testing, so it was ‘normalized’ to each individual’s physical capabilities, rather than given as absolute levels of force required. To minimize fatigue, effort expenditure was equally distributed across both hands. Further, on 25% of the accepted trials, patients were exempt from squeezing and allocated the reward at no cost.

With six levels of reward and effort, 36 possible offer types were available (Fig. 1A, bottom). Each offer type was sampled five times, giving a total of 180 trials divided into five blocks of 36 trials. Trial order was pseudo-randomized, ensuring all patients were presented the offers in the same order. Before the experiment, each participant practiced squeezing the handheld device at each effort level and completed a full practice block with 36 trials. The extracted behavioural parameters were choice (i.e. accept or reject), reaction time and force metrics.

### Imaging

Images were acquired in a 3 T Siemens Verio scanner at the John Radcliffe hospital, Oxford. The neuroimaging protocol included: T_1_-weighted sequence (acquisition time 4 min 54 s, repetition time 2000 ms, echo time 1.94 ms, inversion time 880 ms, flip angle 8°, voxel size 1.0 mm isotropic); a fluid attenuated inversion recovery, or T_2_-FLAIR, sequence (acquisition time 4 min 32 s, repetition time 9000 ms, echo time 88 ms, inversion time 2500 ms, flip angle 150°, voxel size 1.0 × 1.0 × 3.0 mm); and a diffusion weighted echo-planar (EPI) sequence (acquisition time 4 min 40 s, repetition time = 8000 ms, echo time = 86.0 ms, voxel size = 2 mm isotropic, 30 scans with b-value = 1500 s/mm^2^, b = 0 s/mm^2^).

### Analyses

#### Clinical measures

Pearson correlations were used to establish relationships between clinical measures of interest. These were conducted using the ‘cor’ function in R in the ‘stats’ package (https://www.R-project.org/).

#### Raw behavioural data

All primary analyses were conducted using continuous variable measures rather than clinical cut-offs. This was to avoid dichotomizing continuous variables.^61^^,^^62^ Where necessary, clinical cut-offs were used to support primary analyses. Robust-linear regressions were used to assess the relationship between overall acceptance rate and apathy using the lm_robust function in the ‘estimatr’ R package (https://CRAN.R-project.org/package = estimatr). This applied a weighting function to reduce the influence of outlier data and clustering methods to overcome heteroscedasticity. In-depth analysis of behavioural mechanisms was conducted using a logistic regression with mixed effects for the choice data. This was conducted in MATLAB using the *fitglme* function. Trials whose reaction time was <0.4 s were discarded as being accidental, as were those with reaction times > 3 times the standard deviation for each participant where they were likely to have been distracted or inattentive. The total number of excluded trials represented only ∼2% of trials overall. The model included a random effect of participant and fixed effects of reward, effort, and apathy status. All interactions between reward, effort and apathy were included. For each model, the parameter estimate, F-statistic and *P*-value was reported. Statistical significance was inferred when *P*-values were < 0.05 and corrected for multiple comparisons where appropriate. In all analyses, the effort penalty term was squared, in line with motor control principles which assign a quadratic relationship between force requirements and perceived effort costs.^63^^-^^65^

#### Hierarchical drift-diffusion modelling of effort-based decision making

**Figure 2:**
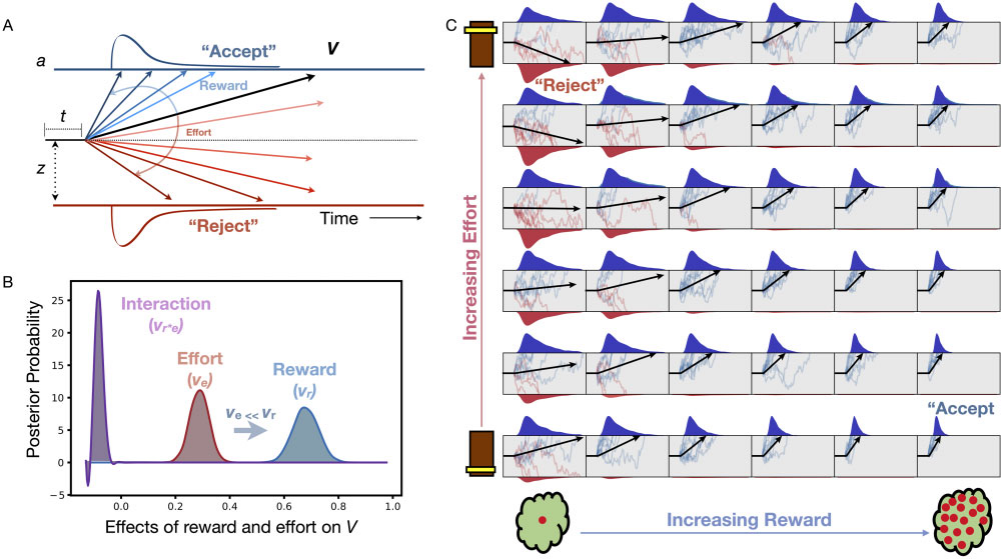
**DDM of effort-based decision making for reward.** (**A**) Model assumes decisions to accept offers arise from a noisy process of evidence accumulation up to a decision boundary, *a*, at a drift rate, *V* and starting point of evidence accumulation termed the bias, *z*. The final term accounts for non-decision time components, *t*. (**B**) Both reward and effort significantly alter drift rate but reward has a significantly greater impact than effort. (**C**) Overview of how drift rate (black arrow) varies with each unique combination of reward and effort posed by our task. The drift rate incrementally rises with increasing reward (*left* to *right*), and falls with increasing effort (from *bottom* to *top*).

Performance on the effort-based decision-making paradigm can be viewed as a two-alternative forced choice (2AFC) task where participants must choose between accepting or rejecting an offer. Framing the task this way enabled us to use well-validated 2AFC modelling approaches such as DDM.^50^^,^^51^^,^^66^ DDM assumes that evidence accumulates in a noisy manner until a decision boundary is reached, at which point a decision is made (i.e. reject or accept). The model is generally described by four parameters: (i) the threshold, *a*, representing the distance between the two alternative decisions (i.e. Accept or reject); (ii) the bias, *z*, representing an *a priori* starting point of evidence accumulation as a function of a where a non-biased starting point = 0.5; (iii) the non-decision time, *t*, which represents time allocated for decision independent processes such as perception, movement initiation and execution; and (iv) the drift rate, *V*, representing the rate of evidence accumulation. This increases as participants accept more offers and reduce their decision times (Fig. 2A).

To accommodate the structure of the experimental task, *a*, *z* and *t* were fixed across conditions, whereas drift rate was varied with the reward and effort in each segment of our decision space (Fig. 2). For each subject the drift rate composed of four subcomponent parameters: (i) the average drift rate across all trials *v*_0_; (ii) the effect of reward on drift rate, or *v*_r_; (iii) the effect of effort on drift rate, or *v*_e_; and (iv) the effect of the reward × effort interaction on drift rate, or *v*_r*e_

The actual drift rate (*V_r_*_,_*_e_*) for each segment within the decision space was therefore assumed to be a linear combination of reward, effort, and their interaction such that:

(1) $$V_{r,e}\;=\;v_{0}+v_{r}-v_{e}-v_{r*e}$$

Overall group acceptance rates (AR) and decision times (DT) were subsequently predicted using the following two equations (see Bogacz *et al*.^66^ for mathematical derivations):

(2) $$\mathrm{AR}\;=\;\frac{1}{1+e^{-2V_{r,e}\;a/c^{2}}}\;$$

(3) $$DT\;=\;\left|\frac{a}{V_{r,e}}\tanh\left(\frac{V_{r,e}*a}{c^{2}}\right)\right|$$

Where *c* represents a noise parameter (set as 1 by default). We conducted a population level analysis with ‘subject’ included as a hierarchical factor, followed by separate fits for each subject. This gave rise to whole group and per subject posterior distributions for *V* (including its subcomponents), *z*, *a* and *t*. Bayesian inference was used to compare the effects of reward and effort on overall drift rate across the whole group. This is reported as the posterior probability (*P*_P__**|**__D_) of our hypothesis of interest. Additionally, for all seven parameters, we extracted the mean of the posterior distribution per participant and assessed their relationship to apathy and depression in robust multiple regressions.

A well-validated DDM toolbox^52^ was used to fit this model to the data (http://ski.clps.brown.edu/hddm_docs/; version 0.6.0; Python 2.7). Informative group mean priors created by Weicki and colleagues were used (see the supplementary material in Wiecki *et al*.^52^) to roughly match parameter values reported in the literature.^67^ Markov chain Monte Carlo methods (MCMC) generated samples from the joint posterior distributions of all model parameters. Six MCMC chains were run, each having 10 000 samples with the first 1000 samples discarded as ‘burn-ins’. The model assumed a 5% fixed probability of outlier reaction time data. Convergence checks were conducted by visualizing trace plots and computing R-hat, or Gelman Ruben, statistics across the six MCMC chains. Posterior predictive checks were conducted to ensure model predictions could accurately retrieve behavioural patterns in the original dataset. A detailed explanation of these model evaluation steps can be found in the Supplementary material, ‘Drift diffusion model evaluation’ section.

#### Structural image preprocessing and analysis

**Figure 3:**
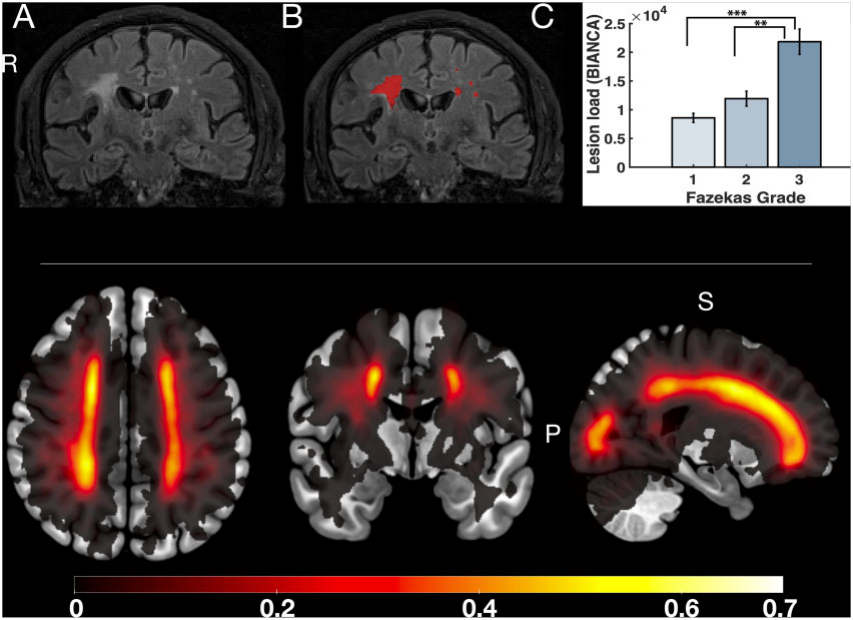
**Quantification of disease severity using white matter lesion load.** *Top*: A representative FLAIR sequence before and after applying BIANCA, an automated classification algorithm, to detect white matter lesions (**A** and **B**, respectively). Lesion load was calculated by applying a threshold of 80% to minimize false positives (**B**). This measure was validated against clinician-scored Fazekas gradings, and demonstrated significant increases in lesion load with higher Fazekas scores (**C**). *Bottom*: Group lesion heat map representing probability of white matter lesion presence (see colour bar) across the whole patient group. Axial, coronal and sagittal views from *left* to *right*.

All MRI images were initially processed and formatted into standardized brain imaging data structure (BIDS).^68^ White matter lesions were classified using a fully automated, supervised method known as BIANCA (brain intensity abnormality classification algorithm).^69^ For each image voxel, BIANCA assigns a probability of there being a white matter lesion of presumed vascular origin. Doing so relies on comparisons with manually segmented training datasets. In this case, we used a training dataset of 18 consecutive patients from the OXVASC study who had recently experienced a TIA or minor stroke. This patient group, described in detail by Sundaresan *et al*.^70^) underwent an identical MRI scanning procedure to our patient cohort and were demographically similar to our patient group. Each scan was manually checked to ensure appropriate classification of white matter lesions by a clinician (Fig. 3A and B). Finally, total lesion load for each participant was extracted based on the number of voxels with a >80% probability of being a lesion. Cross validation of this method was conducted by comparing mean lesion load values based on clinician scored Fazekas grading.^71^ A one way ANOVA confirmed significant increases in lesion load when split by Fazekas score [*F*(1,59) = 22.92, *P*** **<** **0.0001; Fig. 3C]. Specifically, lesion volumes in patients with Fazekas score of 3 were significantly greater than those with grade 2 or 1, respectively. This total lesion load score was subsequently used as one surrogate measure for disease severity in our analyses. Additionally, lacunar infarcts were manually checked by a qualified neurologist and included in our analyses. These were defined based on the STRIVE criteria^5^ as round or ovoid subcortical, fluid filled cavities between 3 mm and 15 mm, which had a white rim on FLAIR MRI.

#### Diffusion image preprocessing and analysis

Voxelwise statistical analysis of the FA data was carried out using TBSS (tract-based spatial statistics),^72^ part of FSL.^73^ Sixty-seven FA images were created by fitting a tensor model to the raw diffusion data using FDT, and then brain-extracted using BET.^74^ All participants' FA data were then aligned into a common space using the nonlinear registration tool FNIRT,^75^^,^^76^ which uses a b-spline representation of the registration warp field.^77^ Next, the mean FA image was created and thinned to create a mean FA skeleton, which represents the centres of all tracts common to the group. Each participant's aligned FA data were then projected onto this skeleton and the resulting data fed into voxel wise cross-participant statistics. Each subject’s data were manually checked at each step and no data were excluded because of quality control issues.

The *randomise* function within FSL was used to carry out non-parametric voxel wise analyses using 5000 permutations. A general linear model was used to investigate the positive and negative associations between apathy as a continuous measure with FA while controlling for depression and age. We then repeated this to assess the association with depression as a continuous measure while controlling for apathy and age. Threshold-free cluster enhancement (TFCE) was used to correct for multiple comparisons across space and corrected *P*-values were thresholded at *P*** **<** **0.05.^78^^,^^79^ Alongside the visible markers of SVD (see above and Fig. 3), average FA across all skeletonized tracts was used as another measure of disease severity to ensure that apathy related changes were not due to generalized disease progression. Specifically, we compared these measures to apathy and accounted for their effects when investigating the influence of apathy on specific FA tracts.

### Data availability

Anonymized data are available on request.

## Results

### Apathy but not depression reduces overall offer acceptance

The influence of apathy severity as a continuous variable on the total number of offers accepted overall was assessed using robust linear regressions. Patients with greater apathy (higher AES score) accepted fewer offers overall [*F*(1,79) = 5.167, *P*** ***=*** **0.026; Fig. 1C]. This effect was retained after including depression, age and cognitive impairment as covariates in a multiple regression [*F*(1,74) = 4.51, *P*** ***=*** **0.037]. By contrast, depression alone did not independently influence the total number of offers accepted [*F*(1,81) = 0.0013, *P*** ***=*** **0.97]. Together these findings suggest an independent effect of apathy but not depression on motivated behaviour on this task. We note though that there was a significant positive correlation between apathy and depression *[*Pearson’s *r*(79) = 0.38, *P*** ***=*** **0.0005], so these constructs are clinically related but also dissociable as shown by performance on our task. Additionally both apathy and depression were independently associated with reduced quality of life in a robust multiple regression [*F*(1,73) = 8.15, *P*** ***=*** **0.005 and *F*(1,73) = 10.81, *P*** ***=*** **0.002, respectively].

### Apathetic behaviour driven by altered responsiveness to reward and effort

A logistic regression with mixed effects was performed to examine how apathy severity alters decision making with varying levels of reward and effort. Two variations of this model were constructed, one using the AES as a continuous variable, and another using the clinical cut-off of AES >34 (Supplementary material, Models 1 and 2 in the ‘Choice models’ section). Model 1 provided a superior fit to the data, as measured by the Bayesian information criterion (BIC_M1_ = 7341 and BIC_M2_ = 7423) and was therefore deferred to where results between the two models diverged.

**Figure 4:**
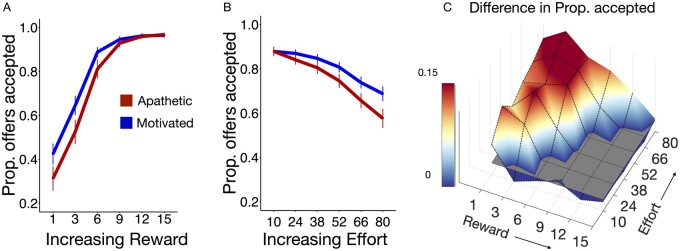
**Behavioural performance comparing apathetic versus motivated SVD patients.** (**A**) Patients with apathy accepted fewer offers when the reward was low and (**B**) effort was high. (**C**) Difference in offers accepted between non-apathetic and apathetic individuals in reward-effort decision space. Data are mean ± standard error (**A** and **B**).

The results for Model 1, visualized in Fig. 4, demonstrated a significant two-way interaction between apathy severity and reward [*t*(14599) = +0.23, *F*(1,14599) = 13.49, *P*** ***=*** **0.0002; Fig. 4A]. Patients with apathy were less responsive to low levels of reward. Additionally, a significant two-way interaction was observed between apathy and effort: more apathetic patients accepted fewer offers at high effort [*t*(14599) = +0.24, *F*(1,14599) = 25.58, *P*** **<** **0.0001; Fig. 4B]. Both these effects were observed in Model 2. Notably there was a discrepancy between the two models. Model 2, which divided patients into two groups (apathetic and motivated), showed a significant three-way interaction between reward, effort and apathy [*t*(14599) = +0.11, *F*(1,14599) = 5.45, *P*** ***=*** **0.019]. In contrast, Model 1, which investigated apathy scores as a continuous variable, did not show such a significant three way interaction [*t*(14248) = +0.085, *F*(1,14248) = 3.54, *P*** ***=*** **0.059]. In this case we deferred to Model 1 as this provided a significantly improved model fit and was less likely to demonstrate false positive statistical results, which are more common when dichotomizing patients into binary subgroups.

### Speed and offer acceptance are explained by evidence accumulation model

In addition to choice, another behavioural parameter of interest was reaction time, which we incorporated alongside the choice data into a single DDM to gain further insight into the behavioural mechanisms of apathy. A DDM^51^ was fitted to the data using Bayesian statistical methods. This approach confers the benefit of providing probability distributions as a measure of certainty in our estimates (components shown in Fig. 2A). Reward increased and effort decreased drift rate respectively (Fig. 2B). A schematic representation of how the drift rate varies with reward and effort manipulations is depicted in Fig. 2C. Note that the magnitude of change incurred by reward on drift rate was significantly larger than that by effort (*P*_P__**|**__D_ ≈ 1). This suggests that overall reward had a larger effect on decisions than effort.

**Figure 5:**
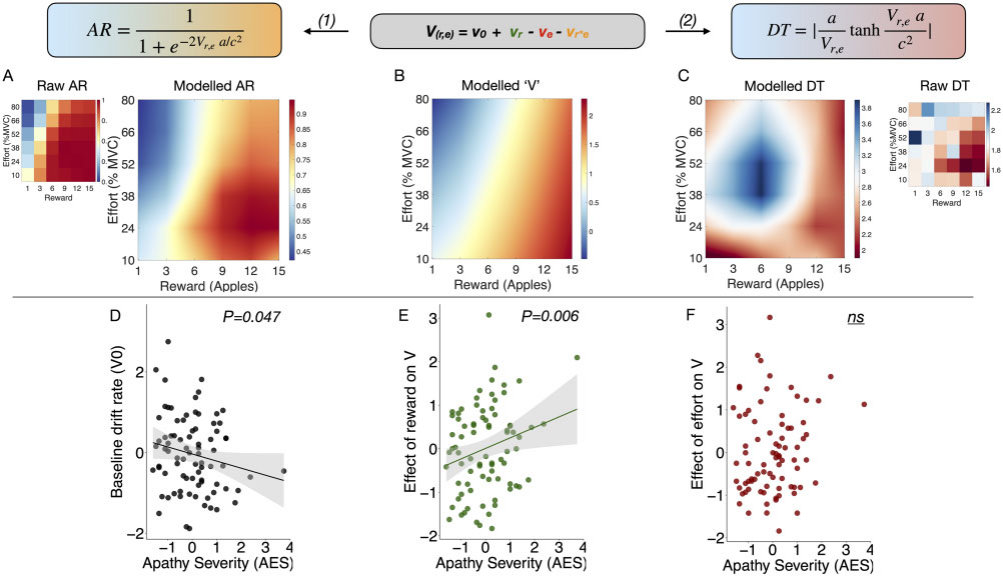
**Predicting apathetic behaviour using model parameters.** *Top*: Model estimates of drift rate in each sector of decision space accurately predicts the acceptance rate (AR) when compared with the raw choice data (**A**). (**C**) Similarly, the model accurately predicts reaction times across different reward-effort combinations. Both acceptance rate and reaction time are derived from the drift rate, *V_r_*_,_*_e_* (**B**) using equations (1) and (2), respectively. Apathy severity (given here as *z*-scores) is associated with reduced drift rate overall (**D**), and specifically altered (change) the effect of reward on drift rate (**E**). (**F**) Apathy did not significantly influence the effect of effort on the drift rate.

The model predictions for both acceptance rates and decision times were qualitatively similar to the raw data (Fig. 5, top). Notably, the model accurately predicted the pattern of increasing acceptance rates as the reward increased (Fig. 5A) as well as the high rejection zones (Fig. 5A) where the reward was low and/or the effort was high. Similarly, the segments of decision space with the highest decision speed (Fig. 5C) were also accurately predicted. Both acceptance rates and decision times were derived from the drift rate, *V*, using the equations highlighted at the top of Fig. 5. The seven model parameters were subsequently regressed against apathy and depression using robust multiple regressions.

### Apathy associated with reduced evidence accumulation (drift) rate

Age was included as a covariate, as it independently decreased the baseline drift rate, *v*_0_, [*F*(1,80) = 4.97, *P*** **=** **0.028]. Crucially, apathy was associated with significantly reduced *v*_0_, [*F*(1,76) = 4.08, *P*** **=** **0.047; Fig. 5D] while accounting for depression, which had no effect on drift rate [*F*(1,76) = 0.11, *P*** ***=*** **0.73]. Additionally, apathy significantly altered the effect of reward on drift rate, *v*_r_ [*F*(1,76) = 7.47, *P*** **=** **0.006; Fig. 5E], but did not influence the effects of effort on drift rate, *v*_e_, or the reward × effort interaction with drift rate, *v*_r*e_ [*F*(1,76) = 1.53, *P*** **=** **0.22; Fig. 5F; and *F*(1,76) = 1.59, *P*** **=** **0.21, respectively]. Finally, apathy was not associated with changes to the decision threshold boundary, *a* [*F*(1,76) = 0.93, *P*** **=** **0.33], nor the bias, *z* [*F*(1,76) = 0.15, *P*** ***=*** **0.70].

### Depression associated with increase in evidence required to make decisions

While accounting for apathy and age, depression was associated with an increase in the decision boundary, *a* [*F*(1,76) = 4.74, *P*** ***=*** **0.033]. Depression was not associated with drift rate components *v*_r_, *v*_e_, or *v*_r*e_ [*F*(1,76) = 2.62, *P*** **=** **0.11; *F*(1,76) = 0.066, *P*** **=** **0.80; and *F*(1,76) = 0.62, *P*** **=** **0.43, respectively]. Finally there was no association between depression and the bias term, *z* [*F*(1,76) = 2.67, *P*** ***=*** **0.098].

Taken together, this analysis demonstrates that apathy and depression have dissociable effects on DDM parameters. Namely, apathy severity reduced the rate of evidence accumulation (drift rate) towards accepting offers. Additionally, the most important criterion in driving decisions in apathy was reward magnitude. On the other hand, depression increased the amount of evidence required for making a decision, resulting in longer reaction times and less noisy decisions, likely due to increased caution when weighing up offers.

It might be argued that these effects relating apathy to drift rate and its components are due to disease severity rather than apathy *per se*. To investigate this, we included white matter lesion load and global white matter integrity (average FA) as covariates in an additional model. Both the association of apathy with *v*_0_ and *v*_r_ were retained [*F*(1,56) = 4.12, *P*** ***=*** **0.047; and *F*(1,56) = 5.43, *P*** ***=*** **0.02, respectively]. Similar results were obtained if only one of white matter lesion load or global white matter integrity was used as a covariate instead of both [*F*(1,57) = 4.51, *P*** ***=*** **0.038; and *F*(1,57) = 4.12, *P*** ***=*** **0.047, respectively]. Hence general disease severity alone does not account for the behavioural changes associated with apathy in the DDM. Together, the findings suggest that apathetic patients require more time to accumulate evidence in favour of accepting offers. This effect is particularly evident when evaluating rewards, hence their tendency to reject low reward offers more readily (Fig. 4A). Notably, these effects were not observed in depression, nor were they fully explained by disease severity.

### Diffusion-weighted imaging

#### Apathy characterized by reduced white matter integrity

**Figure 6:**
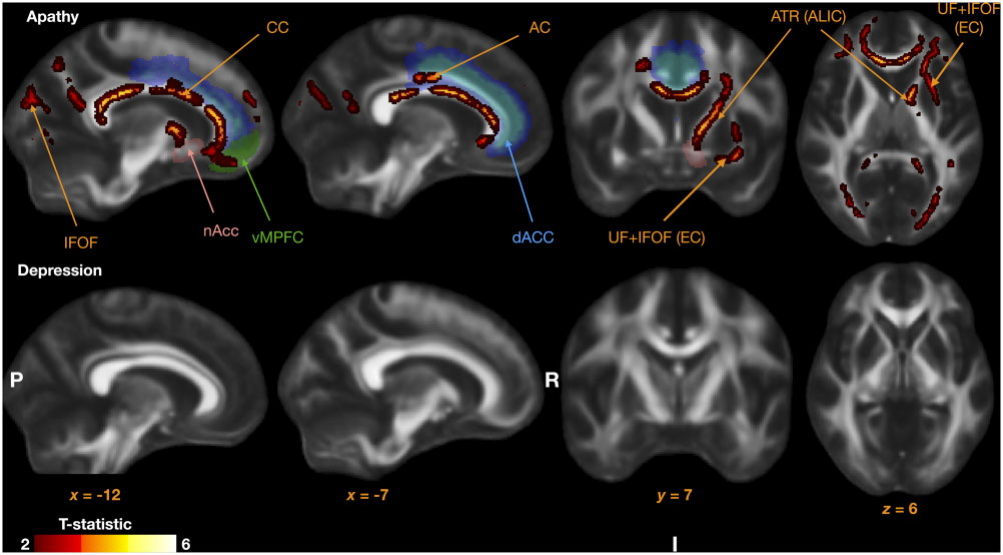
**Tracts with reduced fractional anisotropy associated with apathy.** Relevant brain regions are highlighted to demonstrate tract positions relative to the dorsal ACC (dACC, blue), vMPFC, and the nucleus accumbens (nAcc), all implicated in effort based decision-making studies in healthy individuals. Note no regions were identified to be associated with depression (*bottom*). AC = anterior cingulum; ALIC = anterior limb of the internal capsule; ATR = anterior thalamic radiation; EC = external capsule; IFOF = inferior fronto-occipital fasciculus; UF = uncinate fasciculus.

Sixty-seven patients were included in this analysis. FA was negatively associated with apathy within several white matter tracts (Fig. 6). These included: anterior cingulum bilaterally, anterior thalamic radiation within the anterior limb of the internal capsule (left), corpus callosum, and uncincate/inferior fronto-occipital fasciculus within the external capsule.

The association between FA in these tracts and apathy was retained after adjusting for both lesion load and the number of lacunar infarcts in a multiple regression (Supplementary material). Neither lesion load nor the number of lacunar infarcts was primarily associated with apathy [*F*(1,59) = 0.098, *P*** ***=*** **0.75; and *F*(1,59) = 0.1, *P*** ***=*** **0.75, respectively]. No positive association between FA and apathy was observed in any tract. Importantly, depression was not associated with any changes in FA after accounting for apathy.

Additionally, we extracted the average FA across the whole white matter tract skeleton for each subject to investigate the association between apathy and global white matter integrity. When the same covariates as in the initial analysis were included, there were no significant associations between average FA and either apathy [*F*(1,63) = 1.29, *P*** ***=*** **0.26] or depression [*F*(1,63) = 0.32, *P*** ***=*** **0.57]. However, age was significantly associated with reductions in FA [*F*(1,63) = 11.20, *P*** ***=*** **0.0014] confirming its generalized effect on white matter integrity. Thus, the effects of apathy on white matter were not general but instead specific to those identified in Fig. 6.

#### Drift rate is significantly associated with reductions in fractional anisotropy

**Figure 7:**
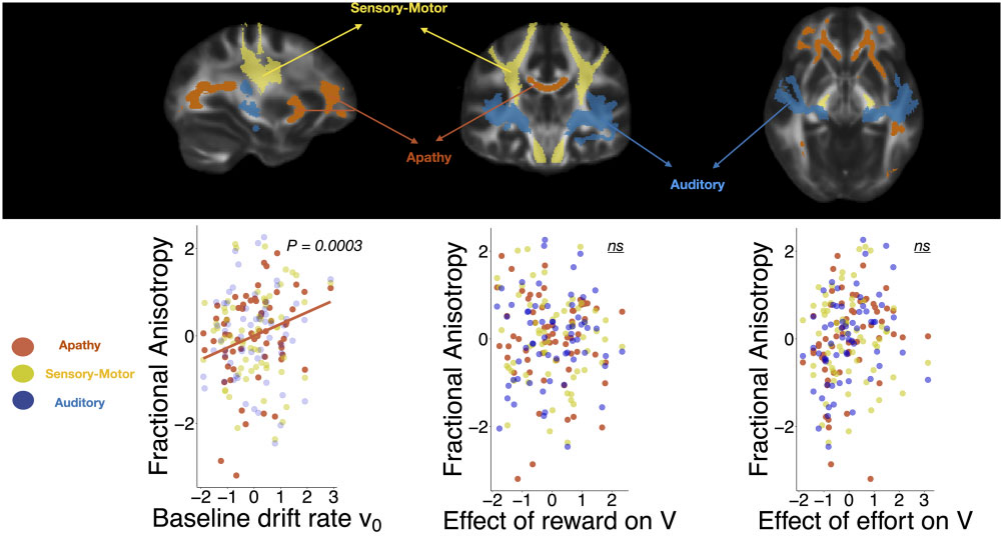
**Drift rate is associated with white matter integrity in the apathy network.** Association between drift diffusion model parameters and fractional anisotropy within the apathy network (*top*, red) and two control networks (sensory-motor, green and auditory, blue). Drift rate was significantly associated with white matter integrity within the apathy network (*bottom left*) but not the control networks. These effects were not associated with the reward and effort related changes in drift rate.

Is drift rate in the DDM associated with white matter integrity changes within the brain network identified as being associated with apathy? To answer this question the FA values within this network (Fig. 7, top) were extracted, together with those in two ‘control’ white matter tract networks. These were the sensorimotor network (SMT), consisting of the corticospinal tracts and superior longitudinal fasciculi bilaterally (Fig. 7, top), and the auditory network, consisting of the acoustic radiations bilaterally (Fig. 7, top).^80^ There was a significant positive correlation between baseline drift rate *v*_0_ and FA within the apathy network, but not the two control networks (Fig. 7, bottom left), even after correcting for multiple comparison [*F*(1,58) = 14.58, *P*** ***=*** **0.0009] indicating that integrity of those tracts is associated with higher rate of evidence accumulation to accept offers.

No significant associations were observed between FA in the apathy network and the effects of reward [*F*(1,58) = 1.15, *P*** ***=*** **0.29] or effort [*F*(1,58) = 0.058, *P*** ***=*** **0.81] on drift rate. There was a significant positive association between the effect of effort on drift rate and the auditory network but this did not survive correction for multiple comparison. The decision threshold and bias parameters were also not associated with FA changes [*F*(1,58) = 1.64, *P*** ***=*** **0.20; and *F*(1,58) = 0.95, *P*** ***=*** **0.33, respectively]. Overall, this analysis demonstrates that significant decreases in motivated behaviour, as defined by a general reduction in baseline drift rate, are associated with white matter tract changes identified to be associated with clinical apathy in SVD. Notably, this was observed for only the overall drift rate, and not the interactions of reward and effort with drift rate. This suggests that while the overall drift rate reductions in apathy are associated with white matter integrity changes, the altered reward responsiveness in apathetic subjects may arise via secondary mechanisms.

## Discussion

The findings presented here demonstrate that apathy in late-onset, sporadic SVD is characterized by distinct structural and functional correlates. SVD patients with apathy accepted less monetary offers overall in an effort-based decision-making task, and this effect was found to be independent of depression severity. Additionally, further behavioural analyses suggest apathy is mediated by a combination of reduced sensitivity to reward (reduced acceptance of offers at low rewards) and increased aversion to high effort (reduced acceptance of offers at high efforts). Combining both choice and reaction time data into a mechanistic model of decision-making (DDM), revealed two key signatures of apathetic behaviour that were independent from depression. These were a significant reduction in the rate at which individuals accumulate evidence in favour of initiating rewarded effortful actions (decreased *v_0_*), as well as an increased reliance of this drift rate on reward magnitude. Static properties of the DDM (non-decision time, initial bias, threshold) were unaffected by apathy. Importantly, these effects were dissociated from depression, which increased the overall evidence required for making decisions, represented by an increase in the decision boundary parameter.

Reduced white matter tract integrity, which crucially could not be accounted for by general SVD disease severity, was associated with apathy. This included tracts within the cingulum and the anterior limb of the internal capsule which are known to connect brain regions commonly implicated in effort-based decision making such as the ACC, vmPFC and ventral striatum.^27^^,^^28^^,^^33^ By contrast, no significant white matter changes were associated with depression when accounting for apathy. Finally, by using task-derived DDM parameters, we were able to associate the behavioural and anatomic changes in apathy. There was a significant negative correlation between drift rate to decision threshold and the brain network identified to be associated with apathy in SVD, but not two control networks. Together, these findings reveal a strong association between structural and functional changes underlying the syndrome of apathy in SVD.

### Behavioural findings and modelling

The behavioural effects delineated here were associated with apathy, rather than arising as a general consequence of SVD severity (indexed by white matter lesion load or average FA), cognitive impairment, age and—most importantly—depression. Thus, although apathy and depression were significantly correlated on clinical measures, they diverged with respect to their association with performance on the effort-based decision-making task used here, as well as in the neuroimaging findings (discussed in detail below).

The behaviour of apathetic patients with sporadic SVD on our paradigm in some ways resembles that previously reported in both CADASIL^23^ and Parkinson’s disease.^20^^,^^22^ Specifically, individuals with apathy were less likely to accept offers whose reward outcome was low (Fig. 4A), despite engaging with high reward offers in a similar manner as their motivated counterparts. On the other hand, we also observed an increased rejection of offers by SVD apathetic participants when effort requirements were high (Fig. 4B), which has not been reported previously in CADASIL^23^ or Parkinson’s disease.^20^^,^^22^ This suggests that while reward insensitivity might be a common, unifying behavioural finding in apathy across neurological conditions, there may also be some differences in effort-related decisions when comparing behaviour across patient groups. Apathy in sporadic SVD shows evidence of significant hypersensitivity to effort, as well as lack of incentivization by low rewards.

Choice data alone are an incomplete representation of motivated behaviour as they do not include reaction time metrics. We therefore combined both into a Bayesian DDM, providing for the first time a mechanistic account of apathetic behaviour using this framework. Effort-based decisions could be described using seven core parameters. Two of these were altered in apathy while accounting for depression (Fig. 5, bottom). First, the baseline drift rate (*v*_0_) was reduced suggesting that evidence accumulation towards making a choice is reduced overall in apathy (Fig. 5D). Second, reward was significantly more likely to alter the drift rate in apathetic patients compared to their motivated counterparts (Fig. 5E). This can be interpreted as low reward promoting more rejection, without changing the speed or efficiency of decision-making when reward is high. Drift rate changes can be mapped on to changes in signal-to-noise ratio of information accumulation. However, our results cannot be explained solely in terms of increased decision noise in apathy, because crucially this would lead to differences in both effort and reward sensitivity. Additionally, we demonstrated this empirically by including decision noise as a covariate in an additional drift diffusion model, which demonstrated no association between apathy and decision noise (Supplementary material). Note that it is not possible to separate global changes in decision noise from changes in threshold, in the standard DDM. Taken together, these findings suggest that while apathetic patients may be slower and less likely to exert effort overall, their willingness to exert effort is significantly influenced by how rewarding the outcome of their action is. By contrast, depression was independently associated with an increase in the threshold boundary for making decisions, demonstrating dissociable behavioural effects of apathy and depression in SVD.

To the best of our knowledge, this is the first time it has been shown that performance on this effort-based decision making task can be accurately and successfully modelled using DDM^52^ and how it relates specifically to a significant reduction in the drift rate in apathy. DDM is now a well validated approach, traditionally used to model perceptual paradigms^51^ and more recently, value-based decisions.^81^^-^^83^ One important potential use of DDM in apathy may be to quantify treatment effects following potential therapeutic interventions, as well as to reveal their precise mechanism of action—on reward or effort sensitivity or decision threshold. A recent study demonstrated that the decision threshold parameter *a* could predict relapses in patients with depression after discontinuation of anti-depressant therapy.^84^ This is notable as we were able to demonstrate a similar association between questionnaire measures of depression and the parameter *a* in our SVD cohort.

In apathy there has been some evidence suggesting a role for dopaminergic therapy as a possible treatment.^19^^,^^31^^,^^85^ Therefore, DDM parameters may serve as valuable behavioural markers of disease progression and treatment response. They can also be used to investigate the cognitive effects of invasive procedures such as deep brain stimulation whose targets are often implicated in effort-based decision making.^86^^-^^88^ Combined with DDM, this task can now also be used to validate amotivated behaviour in other neurological conditions where it is common such as Alzheimer’s disease,^89^ Huntington’s disease,^90^ traumatic brain injury^59^^,^^91^ and stroke.^92^

### Brain networks

Anatomically, apathy was associated with white matter tract changes in several tracts including: anterior cingulum, anterior limb of the internal capsule, corpus callosum, uncincate fasciculus, anterior thalamic radiation and inferior fronto-occipital fasciculus (Fig. 6). Notably, the DDM drift parameter *v_0_*, which represents the baseline rate of approaching the decision threshold (Fig. 2), was also significantly associated with the changes in these tracts even after controlling for two control tracts (Fig. 7). Additionally, apathy was not associated with a global measure of white matter tract integrity, defined as the mean FA across all defined tracts. This suggests that the anatomical changes observed in apathy are specific to the aforementioned tracts and may be associated with overall changes in motivated behaviour as predicted by the modelling parameter *v_0_*. Finally, whereas these changes were observed while accounting for depression, there were no significant white matter associations noted for depression when accounting for apathy (Fig. 6).

The human cingulum bundle is considered to be a large-scale white matter system, carrying fibres associated with several different major white matter tracts, including those linking vmPFC, ACC and the presupplementary to the motor apparatus in supplementary motor and primary motor regions.^93^ It has been linked to the initiation of self- rather than externally-generated movements^93^ and lesions involving the cingulum can result in severe forms of apathy manifest as akinetic mutism.^34^^,^^35^ The finding of cingulum involvement in our study is largely concordant with previous reports in both genetic^23^ and sporadic^6^^,^^24^ forms of SVD. The anterior cingulum bundle also has been previously implicated in apathy across several different neurological conditions,^6^^,^^23^^,^^94^^,^^95^ as well as in healthy individuals.^27^

Functional imaging studies in healthy individuals have led to the conclusion that cortical regions linked by the cingulum play a key role in effort-based decision making for rewards. While the ACC appears to integrate reward and effort signals to compute net subjective value,^27^^,^^28^^,^^33^ activation within vmPFC correlates positively with increasing reward magnitude.^33^^,^^96^ In the current study, only a specific portion of the bundle appeared to be associated with apathy (Fig. 6, top row), in close proximity to the anterior mid-cingulate gyrus, suggesting that only alterations within the cingulum here might be sufficient to alter motivational states. This was supported by a recent study by Caruana and colleagues who investigated the functional localization within the cingulate cortex through intracortical stimulation of 329 subjects.^97^ They observed an exclusive localization of goal-directed behaviour to the anterior mid-cingulate region supporting our findings.

Additionally, the imaging analyses performed here identified the internal limb of the internal capsule as being associated with apathy (Fig. 6, top row). Recent evidence has highlighted an important role of the anterior limb of the internal capsule (ALIC) as a conduit for tracts linking frontal brain regions (including both medial orbitofrontal cortex, vmPFC and dACC) to subcortical structures including the ventral striatum, midbrain and medial thalamic nuclei.^98^^-^^100^ A tracing study by Safadi *et al*.^100^ further segmented tract pathways within the ALIC into five distinct subgroups based on their cortical origins, demonstrating that white matter tract deficits in distinct subsegments within the ALIC. Unsurprisingly, the ALIC has been used as a deep brain stimulation target for several psychiatric conditions, including major depressive disorder^101^ and OCD.^102^

Widespread changes associated with apathy were also observed within the corpus callosum and uncinate fasciculi bilaterally (Fig. 6). Both tracts have previously been associated with apathy in SVD,^6^^,^^23^ as well as Alzheimer’s disease.^103^^,^^104^ The brain regions connected by these tracts (Fig. 6, top left) encompass the medial forebrain regions such as the vmPFC and dACC, as well as subcortical structures within the basal ganglia such as the ventral striatum.^17^^,^^25^^,^^105^

That we were able to correlate these changes to our behavioural modelling parameters supports the hypothesis that apathetic behaviour arises from specific white matter tract changes within a network connecting medial frontal regions to the basal ganglia. However, it is important to note that these brain changes were associated with only the baseline drift rate *v_0_* and crucially were not linked to the reward and effort sensitivities *per se* (Fig. 7). By contrast, DDM revealed also effects of reward on drift rate, *v*: reward, significantly altered drift rate in apathetic compared to motivated patients. The lack of such an association with white matter structural parameters might potentially be due to lack of sensitivity of MRI or current analysis tools. An alternative explanation is that it is possible that some behavioural manifestations of apathy do not have structural correlates. For example, they might occur as a result of alterations of neurotransmitter changes. Regardless of the precise cause, the findings presented here suggest that white matter tract changes reduce overall goal-directed behaviour. This might in turn alter responsiveness to reward outcomes and/or effort costs, but exactly how this occurs remains to be established.

The associations between FA changes and apathy in our study were present after adjusting for lesion load and lacunar infarcts, neither of which were significantly predictive of apathy in our patient group. While some studies have demonstrated such associations between apathy and visible markers of SVD, these findings are often inconsistent.^36^^,^^37^^,^^39^^,^^40^^,^^106^ Moreover, recent findings show that DWI metrics can be more sensitive than visible markers of SVD alone in detecting white matter disruption.^45^^,^^107^^,^^108^

In summary, this is the largest study to date to combine behavioural and anatomical methods to investigate the syndrome of apathy in SVD. We also report the first application of drift diffusion modelling to a well-validated paradigm, which can be easily replicated in future studies. Importantly, parameters from this model were strongly associated with the anatomical changes, allowing investigators to bridge findings across behaviour, modelling and neuroimaging techniques. The findings presented here provide some support for a transdiagnostic model of apathy, across neurological disorders. While the deficits in reward related processing were largely concordant with previous studies, we also report effort aversion in apathy for the first time. Both effects may arise in apathy as a result of network disruption, in this case via white matter tract changes, within the medial frontal regions and the basal ganglia.

## Supplementary Material

awab013_Supplementary_Data

## Abbreviations

ACC: anterior cingulate cortex
AES: Apathy Evaluation Scale
DDM: drift diffusion modelling
FA: fractional anisotropy
SVD: small vessel cerebrovascular disease
vmPFC: ventromedial prefrontal cortex

## References

[1] Van Der Flier WM , Skoog I, Schneider JA, et al Vascular cognitive impairment. Nat Rev Dis Primers. 2018;4:18003. 29446769 10.1038/nrdp.2018.3

[2] Pantoni L. Cerebral small vessel disease: from pathogenesis and clinical characteristics to therapeutic challenges. Lancet Neurol. 2010;9:689-696. 20610345 10.1016/S1474-4422(10)70104-6

[3] Prins ND , Scheltens P. White matter hyperintensities, cognitive impairment and dementia: An update. Nat Rev Neurol. 2015;11:157-165. 25686760 10.1038/nrneurol.2015.10

[4] Ter Telgte A , Van Leijsen EMC, Wiegertjes K, et al Cerebral small vessel disease: from a focal to a global perspective. Nat Rev Neurol. 2018;14:387-398. 29802354 10.1038/s41582-018-0014-y

[5] Wardlaw JM , Smith EE, Biessels GJ, et al Neuroimaging standards for research into small vessel disease and its contribution to ageing and neurodegeneration. Lancet Neurol. 2013;12:822-838. 23867200 10.1016/S1474-4422(13)70124-8 PMC3714437

[6] Hollocks MJ , Lawrence AJ, Brookes RL, et al Differential relationships between apathy and depression with white matter microstructural changes and functional outcomes. Brain 2015;138:3803-3815. 26490330 10.1093/brain/awv304 PMC4655344

[7] Kazui H , Yoshiyama K, Kanemoto H, et al Differences of behavioral and psychological symptoms of dementia in disease severity in four major dementias. PLoS One. 2016;11:e0161092. 27536962 10.1371/journal.pone.0161092 PMC4990196

[8] Clarke DE , Van Reekum R, Simard M, et al Apathy in dementia: an examination of the psychometric properties of the apathy evaluation scale. J Neuropsychiatry Clin Neurosci. 2007;19:57-64. 17308228 10.1176/jnp.2007.19.1.57

[9] Tierney SM , Woods SP, Weinborn M, et al Real-world implications of apathy among older adults: independent associations with activities of daily living and quality of life. J Clin Exp Neuropsychol. 2018;40:895-903. 29532707 10.1080/13803395.2018.1444736

[10] Eurelings LSM , van Dalen JW, ter Riet G, et al Apathy and depressive symptoms in older people and incident myocardial infarction, stroke, and mortality: a systematic review and meta-analysis of individual participant data. Clep. 2018;10:363-379. 10.2147/CLEP.S150915 PMC5894652 29670402

[11] Feast A , Orrell M, Charlesworth G, et al Behavioural and psychological symptoms in dementia and the challenges for family carers: systematic review. Br J Psychiatry. 2016;208:429-434. 26989095 10.1192/bjp.bp.114.153684 PMC4853642

[12] Starkstein SE, Leentjens AFG. The nosological position of apathy in clinical practice. Journal of Neurology, Neurosurgery & Psychiatry. 2008;79:1088-1092. 10.1136/jnnp.2007.136895 18187477

[13] Le Heron C , Holroyd CB, Salamone J, et al Brain mechanisms underlying apathy. J Neurol Neurosurg Psychiatry. 2019;90:302-312. 30366958 10.1136/jnnp-2018-318265 PMC6518466

[14] Walton ME , Bannerman DM, Rushworth MFS. The role of rat medial frontal cortex in effort-based decision making. J Neurosci. 2002;22:10966-11003. 12486195 10.1523/JNEUROSCI.22-24-10996.2002 PMC6758435

[15] Walton ME , Kennerley SW, Bannerman DM, et al Weighing up the benefits of work: behavioral and neural analyses of effort-related decision making. Neural Networks. 2006;19:1302-1314. 16949252 10.1016/j.neunet.2006.03.005 PMC2519033

[16] Salamone JD , Yohn SE, López-Cruz L, et al Activational and effort-related aspects of motivation: neural mechanisms and implications for psychopathology. Brain 2016;139:1325-1347. 27189581 10.1093/brain/aww050 PMC5839596

[17] Husain M , Roiser JP. Neuroscience of apathy and anhedonia: a transdiagnostic approach. Nature Rev Neurosci. 2018;19:470-484. 29946157 10.1038/s41583-018-0029-9

[18] Chong TTJ , Bonnelle V, Manohar S, et al Dopamine enhances willingness to exert effort for reward in Parkinson’s disease. Cortex. 2015;69:40-46. 25967086 10.1016/j.cortex.2015.04.003 PMC4533227

[19] Chong TTJ , Husain M. The role of dopamine in the pathophysiology and treatment of apathy. Prog Brain Res. 2016;229:389-426. 27926449 10.1016/bs.pbr.2016.05.007

[20] Le Heron C , Plant O, Manohar S, et al Distinct effects of apathy and dopamine on effort-based decision-making in Parkinson’s disease. Brain2018c;141:1455-1469 29672668 10.1093/brain/awy110 PMC5917786

[21] Drew DS , Muhammed K, Baig F, et al Dopamine and reward hypersensitivity in Parkinson’s disease with impulse control disorder. Brain 2020;143:2502-2517. 32761061 10.1093/brain/awaa198 PMC7447523

[22] Muhammed K , Manohar S, Husain M. Mechanisms underlying apathy in Parkinson’s disease. Lancet. 2015;385:S71. 26312893 10.1016/S0140-6736(15)60386-5

[23] Le Heron C , Manohar S, Plant O, et al Dysfunctional effort-based decision-making underlies apathy in genetic cerebral small vessel disease. Brain2018b;141:3193-3210. 30346491 10.1093/brain/awy257 PMC6202575

[24] Tay J , Lisiecka-Ford DM, Hollocks MJ, et al Network neuroscience of apathy in cerebrovascular disease. Prog Neurobiol. 2020;188:101785. 32151533 10.1016/j.pneurobio.2020.101785

[25] Pessiglione M , Vinckier F, Bouret S, et al Why not try harder? Computational approach to motivation deficits in neuro-psychiatric diseases. Brain 2018;141:629-650. 29194534 10.1093/brain/awx278

[26] Bartra O , McGuire JT, Kable JW. The valuation system: a coordinate-based meta-analysis of BOLD fMRI experiments examining neural correlates of subjective value. NeuroImage. 2013;76:412-427. 23507394 10.1016/j.neuroimage.2013.02.063 PMC3756836

[27] Bonnelle V , Manohar S, Behrens T, et al Individual differences in premotor brain systems underlie behavioral apathy. Cerebral Cortex. 2016;26:807-819. 26564255 10.1093/cercor/bhv247 PMC4712805

[28] Croxson PL , Walton ME, O'Reilly JX, et al Effort-based cost-benefit valuation and the human brain. J Neurosci. 2009;29:4531-4541. 19357278 10.1523/JNEUROSCI.4515-08.2009 PMC2954048

[29] Kroemer NB , Guevara A, Ciocanea Teodorescu I, et al Balancing reward and work: Anticipatory brain activation in NAcc and VTA predict effort differentially. Neuroimage. 2014;102:510-519. 25108181 10.1016/j.neuroimage.2014.07.060

[30] Schmidt L , Lebreton M, Cléry-Melin ML, et al Neural mechanisms underlying motivation of mental versus physical effort. PLoS Biol. 2012;10:e1001266. 22363208 10.1371/journal.pbio.1001266 PMC3283550

[31] Adam R , Leff A, Sinha N, et al Dopamine reverses reward insensitivity in apathy following globus pallidus lesions. Cortex. 2013;49:1292-1303. 22721958 10.1016/j.cortex.2012.04.013 PMC3639369

[32] Manohar SG , Husain M. Human ventromedial prefrontal lesions alter incentivisation by reward. Cortex. 2016;76:104-120. 26874940 10.1016/j.cortex.2016.01.005 PMC4786053

[33] Skvortsova V , Palminteri S, Pessiglione M. Learning to minimize efforts versus maximizing rewards: computational principles and neural correlates. J Neurosci. 2014;34:15621-15630. 25411490 10.1523/JNEUROSCI.1350-14.2014 PMC6608437

[34] Barris RW , Schuman HR. Bilateral anterior cingulate gyrus lesions: syndrome of the anterior cingulate gyri. Neurology 1953;3:44. 13013498 10.1212/wnl.3.1.44

[35] Németh G , Hegedüs K, Molnâr L. Akinetic mutism associated with bicingular lesions: clinicopathological and functional anatomical correlates. Eur Arch Psychiatry Neurol Sci. 1988;237:218-222. 2849547 10.1007/BF00449910

[36] Grool AM , Geerlings MI, Sigurdsson S, et al Structural MRI correlates of apathy symptoms in older persons without dementia: AGES-Reykjavik Study. Neurology 2014;82:1628-1635. 24739783 10.1212/WNL.0000000000000378 PMC4013817

[37] Grool AM , Gerritsen L, Zuithoff NPA, et al Lacunar infarcts in deep white matter are associated with higher and more fluctuating depressive symptoms during three years follow-up. Biological Psychiatry. 2013;73:169-176. 23079234 10.1016/j.biopsych.2012.08.024

[38] Kim HJ , Kang SJ, Kim C, et al The effects of small vessel disease and amyloid burden on neuropsychiatric symptoms: A study among patients with subcortical vascular cognitive impairments. Neurobiol Aging. 2013;34:1913-1920. 23414669 10.1016/j.neurobiolaging.2013.01.002

[39] Oudega ML , Siddiqui A, Wattjes MP, et al Are apathy and depressive symptoms related to vascular white matter hyperintensities in severe late life depression? J Geriatr Psychiatry Neurol. 2021;34:21-28. 32036772 10.1177/0891988720901783

[40] Delrieu J , Desmidt T, Camus V, et al Alzheimer's Disease Neuroimaging Initiative, et al Apathy as a feature of prodromal Alzheimer’s disease: an FDG-PET ADNI study. Int J Geriatr Psychiatry. 2015;30:470-477. 24953008 10.1002/gps.4161

[41] Wouts L , Kessel M, Beekman ATF, et al Empirical support for the vascular apathy hypothesis: A structured review. Int J Geriatr Psychiatry. 2020;35:3-11. 31617249 10.1002/gps.5217 PMC6916153

[42] Lawrence AJ , Patel B, Morris RG, et al Mechanisms of cognitive impairment in cerebral small vessel disease: multimodal MRI results from the St George’s cognition and neuroimaging in stroke (SCANS) study. PLoS ONE. 2013;8:e61014. 23613774 10.1371/journal.pone.0061014 PMC3632543

[43] Moonen JEF , Foster-Dingley JC, Van Den Berg-Huijsmans AA, et al Influence of small vessel disease and microstructural integrity on neurocognitive functioning in older individuals: the DANTE study Leiden. AJNR Am J Neuroradiol. 2017;38:25-30. 27659190 10.3174/ajnr.A4934 PMC7963662

[44] Pasi M , Van Uden IWM, Tuladhar AM, et al White matter microstructural damage on diffusion tensor imaging in cerebral small vessel disease: clinical consequences. Stroke. 2016;47:1679-1684. 27103015 10.1161/STROKEAHA.115.012065

[45] Tay J , Tuladhar AM, Hollocks MJ, et al Apathy is associated with large-scale white matter network disruption in small vessel disease. Neurology 2019;92:e1157-1167. 30737341 10.1212/WNL.0000000000007095 PMC6511108

[46] Van Agtmaal MJM , Houben AJHM, Pouwer F, et al Association of microvascular dysfunction with late-life depression: A systematic review and meta-analysis. JAMA Psychiatry. 2017;74:729. 28564681 10.1001/jamapsychiatry.2017.0984 PMC5710252

[47] Ishizaki J , Mimura M. Dysthymia and apathy: diagnosis and treatment. Depress Res Treat. 2011;2011:893905. 21747995 10.1155/2011/893905 PMC3130974

[48] Levy ML , Cummings JL, Fairbanks LA, et al Apathy is not depression. JNP. 1998;10:314-319. 10.1176/jnp.10.3.314 9706539

[49] Starkstein SE , Fedoroff JP, Price TR, et al Apathy following cerebrovascular lesions. Stroke. 1993;24:1625-1630. 8236333 10.1161/01.str.24.11.1625

[50] Ratcliff R. A theory of memory retrieval. Psychol Rev 1978;85:59-108.

[51] Ratcliff R , McKoon G. The diffusion decision model: theory and data for two-choice decision tasks. Neural Comput. 2008;20:873-922. 18085991 10.1162/neco.2008.12-06-420 PMC2474742

[52] Wiecki TV , Sofer I, Frank MJ. HDDM: hierarchical bayesian estimation of the drift-diffusion model in Python. Front Neuroinform. 2013;7:14. 23935581 10.3389/fninf.2013.00014 PMC3731670

[53] Hurford R , Wolters FJ, Li L, et al Prevalence, predictors, and prognosis of symptomatic intracranial stenosis in patients with transient ischaemic attack or minor stroke: a population-based cohort study. Lancet Neurol. 2020;19:413-421. 32333899 10.1016/S1474-4422(20)30079-X PMC7116132

[54] Zamboni G , Griffanti L, Mazzucco S, et al Age-dependent association of white matter abnormality with cognition after TIA or minor stroke. Neurology 2019;93:e272-e282. 31201296 10.1212/WNL.0000000000007772 PMC6656647

[55] Van Swieten JC , Van Gijn J, Hijdra A, et al Grading white matter lesions on CT and MRI: A simple scale. Journal of Neurology, Neurosurgery and Psychiatry. 1990;53:1080-1083. 10.1136/jnnp.53.12.1080 PMC488320 2292703

[56] Wahlund LO , Barkhof F, Fazekas F, et al A new rating scale for age-related white matter changes applicable to MRI and CT. Stroke. 2001;32:1318-1322. 11387493 10.1161/01.str.32.6.1318

[57] Marin RS , Biedrzycki RC, Firinciogullari S. Reliability and validity of the apathy evaluation scale. Psychiatry Res. 1991;38:143-162. 1754629 10.1016/0165-1781(91)90040-v

[58] Andersson S , Krogstad JM, Finset A. Apathy and depressed mood in acquired brain damage: relationship to lesion localization and psychophysiological reactivity. Psychol Med. 1999;29:447-456. 10218936 10.1017/s0033291798008046

[59] Kant R , Duffy JD, Pivovarnik A. Prevalence of apathy following head injury. Brain Inj. 1998;12:87-92. 9483342 10.1080/026990598122908

[60] Topp CW , Østergaard SD, Søndergaard S, et al The WHO-5 well-being index: A systematic review of the literature. Psychother Psychosom. 2015;84:167-176. 25831962 10.1159/000376585

[61] Altman DG , Royston P. The cost of dichotomising continuous variables. Br Med J. 2006;332:1080. 16675816 10.1136/bmj.332.7549.1080 PMC1458573

[62] Royston P , Altman DG, Sauerbrei W. Dichotomizing continuous predictors in multiple regression: A bad idea. Statist Med. 2006;25:127-141. 10.1002/sim.2331 16217841

[63] Morel P , Ulbrich P, Gail A. What makes a reach movement effortful? Physical effort discounting supports common minimization principles in decision making and motor control. PLoS Biol. 2017;15:e2001323. 28586347 10.1371/journal.pbio.2001323 PMC5460791

[64] Shadmehr R , Huang HJ, Ahmed AA. A representation of effort in decision-making and motor control. Curr Biol. 2016;26:1929-1934. 27374338 10.1016/j.cub.2016.05.065 PMC7912535

[65] Todorov E , Jordan MI. Optimal feedback control as a theory of motor coordination. Nat Neurosci. 2002;5:1226-1235. 12404008 10.1038/nn963

[66] Bogacz R , Brown E, Moehlis J, et al The physics of optimal decision making: a formal analysis of models of performance in two-alternative forced-choice tasks. Psychol Rev. 2006;113:700-765. 17014301 10.1037/0033-295X.113.4.700

[67] Matzke D , Wagenmakers EJ. Psychological interpretation of the ex-gaussian and shifted wald parameters: A diffusion model analysis. Psychon Bull Rev. 2009;16:798-817. 19815782 10.3758/PBR.16.5.798

[68] Gorgolewski KJ , Auer T, Calhoun VD, et al The brain imaging data structure, a format for organizing and describing outputs of neuroimaging experiments. Sci Data. 2016;3:160044. 27326542 10.1038/sdata.2016.44 PMC4978148

[69] Griffanti L , Zamboni G, Khan A, et al BIANCA (Brain Intensity AbNormality Classification Algorithm): a new tool for automated segmentation of white matter hyperintensities. NeuroImage. 2016;141:191-205. 27402600 10.1016/j.neuroimage.2016.07.018 PMC5035138

[70] Sundaresan V , Zamboni G, Le Heron C, et al Automated lesion segmentation with BIANCA: impact of population-level features, classification algorithm and locally adaptive thresholding. NeuroImage. 2019;202:116056. 31376518 10.1016/j.neuroimage.2019.116056 PMC6996003

[71] Fazekas F , Kleinert R, Offenbacher H, et al Pathologic correlates of incidental mri white matter signal hyperintensities. Neurology 1993;43:1683-1683. 8414012 10.1212/wnl.43.9.1683

[72] Smith SM , Jenkinson M, Johansen-Berg H, et al Tract-based spatial statistics: voxelwise analysis of multi-subject diffusion data. NeuroImage. 2006;31:1487-1505. 16624579 10.1016/j.neuroimage.2006.02.024

[73] Smith SM , Jenkinson M, Woolrich MW, et al Advances in functional and structural MR image analysis and implementation as FSL. NeuroImage. 2004;23:S208-S219. 15501092 10.1016/j.neuroimage.2004.07.051

[74] Smith SM. Fast robust automated brain extraction. Hum Brain Mapp. 2002;17:143-155. 12391568 10.1002/hbm.10062 PMC6871816

[75] Andersson JLR , Jenkinson M, Smith S. Non-linear registration aka spatial normalisation. FMRIB technical report TRO7JA2. 2007a.

[76] Andersson JLR , Jenkinson M, Smith SM. Non-linear optimisation. FMRIB technical report TR07JA1. 2007b.

[77] Rueckert D , Sonoda LI, Hayes C, et al Nonrigid registration using free-form deformations: Application to breast mr images. IEEE Trans Med Imaging. 1999;18:712-721. 10534053 10.1109/42.796284

[78] Smith SM , Nichols TE. Threshold-free cluster enhancement: Addressing problems of smoothing, threshold dependence and localisation in cluster inference. NeuroImage. 2009;44:83-98. 18501637 10.1016/j.neuroimage.2008.03.061

[79] Winkler AM , Ridgway GR, Webster MA, et al Permutation inference for the general linear model. NeuroImage. 2014;92:381-397. 24530839 10.1016/j.neuroimage.2014.01.060 PMC4010955

[80] Figley TD , Mortazavi Moghadam B, Bhullar N, et al Probabilistic white matter atlases of human auditory, basal ganglia, language, precuneus, sensorimotor, visual and visuospatial networks. Front Hum Neurosci. 2017;11:306. 28751859 10.3389/fnhum.2017.00306 PMC5508110

[81] Mandali A , Weidacker K, Kim SG, et al The ease and sureness of a decision: evidence accumulation of conflict and uncertainty. Brain 2019;142:1471-1482. 30726914 10.1093/brain/awz013

[82] Sugrue LP , Corrado GS, Newsome WT. Choosing the greater of two goods: neural currencies for valuation and decision making. Nat Rev Neurosci. 2005;6:363-375. 15832198 10.1038/nrn1666

[83] Tajima S , Drugowitsch J, Pouget A. Optimal policy for value-based decision-making. Nat Commun. 2016;7:12400. 27535638 10.1038/ncomms12400 PMC4992126

[84] Berwian IM , Wenzel JG, Collins AGE, et al Computational mechanisms of effort and reward decisions in patients with depression and their association with relapse after antidepressant discontinuation. JAMA Psychiatry. 2020;77:513. 32074255 10.1001/jamapsychiatry.2019.4971 PMC7042923

[85] Thobois S , Ardouin C, Lhommée E, et al Non-motor dopamine withdrawal syndrome after surgery for Parkinson’s disease: predictors and underlying mesolimbic denervation. Brain 2010;133:1111-1127. 20237128 10.1093/brain/awq032

[86] Evens R , Stankevich Y, Dshemuchadse M, et al The impact of Parkinson’s disease and subthalamic deep brain stimulation on reward processing. Neuropsychologia. 2015;75:11-19. 25976111 10.1016/j.neuropsychologia.2015.05.005

[87] Schlaepfer TE , Bewernick BH, Kayser S, et al Deep brain stimulation of the human reward system for major depression - Rationale, outcomes and outlook. Neuropsychopharmacol. 2014;39:1303-14 10.1038/npp.2014.28 PMC3988559 24513970

[88] Seymour B , Barbe M, Dayan P, et al Deep brain stimulation of the subthalamic nucleus modulates sensitivity to decision outcome value in Parkinson’s disease. Sci Rep. 2016;6:32509. 27624437 10.1038/srep32509 PMC5021944

[89] Zhao QF , Tan L, Wang HF, et al The prevalence of neuropsychiatric symptoms in Alzheimer’s disease: systematic review and meta-analysis. J Affect Disord. 2016;190:264-271. 26540080 10.1016/j.jad.2015.09.069

[90] Heath CJ , O'Callaghan C, Mason SL, et al A touchscreen motivation assessment evaluated in Huntington’s disease patients and R6/1 model mice. Front Neurol. 2019;10:858. 31447770 10.3389/fneur.2019.00858 PMC6696591

[91] Starkstein SE , Pahissa J. Apathy following traumatic brain injury. Psychiatr Clin North Am. 2014;37:103-112. 24529426 10.1016/j.psc.2013.10.002

[92] Caeiro L , Ferro JM, Costa J. Apathy secondary to stroke: a systematic review and meta-analysis. Cerebrovasc Dis. 2013;35:23-39. 10.1159/000346076 23428994

[93] Maldonado IL , Parente de Matos V, Castro Cuesta TA, et al The human cingulum: from the limbic tract to the connectionist paradigm. Neuropsychologia. 2020;144:107487. 32470344 10.1016/j.neuropsychologia.2020.107487

[94] Zhang Y , Schuff N, Jahng GH, et al Diffusion tensor imaging of cingulum fibers in mild cognitive impairment and Alzheimer disease. Neurology 2007;68:13-19. 17200485 10.1212/01.wnl.0000250326.77323.01 PMC1941719

[95] Zhang Y , Wu J, Wu W, et al Reduction of white matter integrity correlates with apathy in Parkinson’s disease. Int J Neurosci. 2018;128:25-31. 28648111 10.1080/00207454.2017.1347170

[96] Peters J , Büchel C. Neural representations of subjective reward value. Behav Brain Res. 2010;213:135-141. 20420859 10.1016/j.bbr.2010.04.031

[97] Caruana F , Gerbella M, Avanzini P, et al Motor and emotional behaviours elicited by electrical stimulation of the human cingulate cortex. Brain 2018;141:3035-3051. 30107501 10.1093/brain/awy219

[98] Haber SN , Behrens TEJ. The neural network underlying incentive-based learning: implications for interpreting circuit disruptions in psychiatric disorders. Neuron. 2014;83:1019-1039. 25189208 10.1016/j.neuron.2014.08.031 PMC4255982

[99] Lehman JF , Greenberg BD, Mcintyre CC, et al Rules ventral prefrontal cortical axons use to reach their targets: implications for diffusion tensor imaging tractography and deep brain stimulation for psychiatric illness. J Neurosci. 2011;31:10392-10402. 21753016 10.1523/JNEUROSCI.0595-11.2011 PMC3445013

[100] Safadi Z , Grisot G, Jbabdi S, et al Functional segmentation of the anterior limb of the internal capsule: linking white matter abnormalities to specific connections. J Neurosci. 2018;38:2106-2117. 29358360 10.1523/JNEUROSCI.2335-17.2017 PMC5824744

[101] Bergfeld IO , Mantione M, Hoogendoorn MLC, et al Deep brain stimulation of the ventral anterior limb of the internal capsule for treatment-resistant depression. JAMA Psychiatry. 2016;73:456. 27049915 10.1001/jamapsychiatry.2016.0152

[102] Bourne SK , Eckhardt CA, Sheth SA, et al Mechanisms of deep brain stimulation for obsessive compulsive disorder: effects upon cells and circuits. Front Integr Neurosci. 2012;6:29. 22712007 10.3389/fnint.2012.00029 PMC3375018

[103] Damoiseaux JS , Smith SM, Witter MP, et al White matter tract integrity in aging and alzheimer’s disease. Hum Brain Mapp. 2009;30:1051-1059. 18412132 10.1002/hbm.20563 PMC6870688

[104] Hahn C , Lim HK, Won WY, et al Apathy and white matter integrity in Alzheimer’s disease: a whole brain analysis with tract-based spatial statistics. PLoS One. 2013;8:e53493. 23301077 10.1371/journal.pone.0053493 PMC3536751

[105] Le Heron C , Apps MAJ, Husain M. The anatomy of apathy: A neurocognitive framework for amotivated behaviour. Neuropsychologia. 2018a;118:54-67. 28689673 10.1016/j.neuropsychologia.2017.07.003 PMC6200857

[106] Yao H , Takashima Y, Mori T, et al Hypertension and white matter lesions are independently associated with apathetic behavior in healthy elderly subjects: the Sefuri brain MRI study. Hypertens Res. 2009;32:586-590. 19424282 10.1038/hr.2009.65

[107] Auriel E , Edlow BL, Reijmer YD, et al Microinfarct disruption of white matter structure: a longitudinal diffusion tensor analysis. Neurology 2014;83:182-188. 24920857 10.1212/WNL.0000000000000579 PMC4117171

[108] Veldsman M , Tai XY, Nichols T, et al Cerebrovascular risk factors impact frontoparietal network integrity and executive function in healthy ageing. Nat Commun. 2020;11:4340. 32895386 10.1038/s41467-020-18201-5 PMC7477206

