# Supplementary material for: Apathy in small vessel cerebrovascular disease is associated with deficits in effort-based decision making
Source: Brain. 2021 Mar 18;144(4):1247–62. doi: 10.1093/brain/awab013 (PMC8240747; doi:10.1093/brain/awab013)
Supplement: awab013_Supplementary_Data [file awab013_supplementary_data.pdf]

Supplementary section:

Study flow chart:

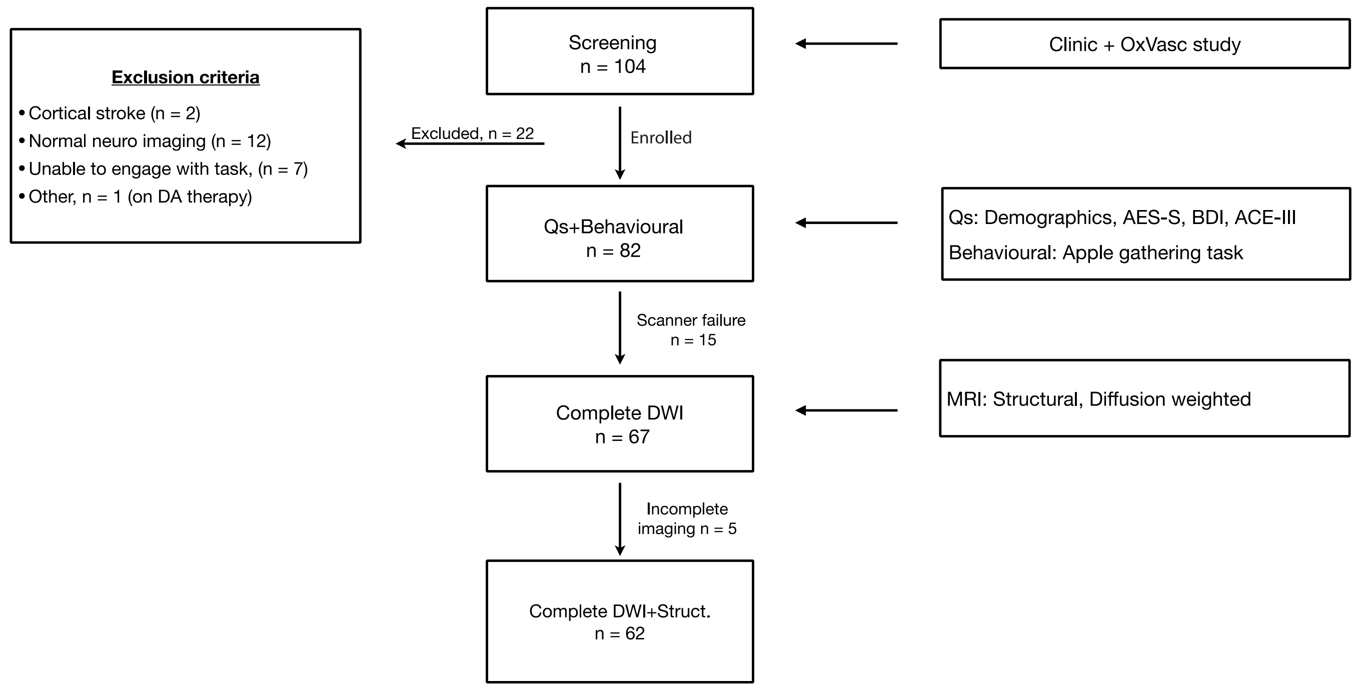

Choice models:

Logistic regressions for raw choice data using a dichotomised cut-off using continuous variable measures as well as a cut off (AES>34) (Model 1 and 2). Key results for each model are in bold within red text boxes. Comparative BIC at the bottom of the model illustration in bold. Models with a lower BIC are favourable. "Apathy_cut" refers to the categorised group (AES>34), and "Apcont" refers to the continuous variable measure.

	**Model 1 (continuous variable)**	**Model 2(cut-off)**
(Intercept)	$\beta = +3.37$<br>$SE = 0.236$<br>$F_{1,14070} = 204.53$<br>**p < 0.0001**	$\beta = +3.44$<br>$SE = 0.24$<br>$F_{1,14421} = 205.10$<br>**p < 0.0001**
Effort	$\beta = +1.18$<br>$SE = 0.0463$<br>$F_{1,14070} = 649.10$<br>**p < 0.0001**	$\beta = +1.18$<br>$SE = 0.0464$<br>$F_{1,14421} = 645.23$<br>**p < 0.0001**
Reward	$\beta = +2.66$<br>$SE = 0.0616$<br>$F_{1,14070} = 1871.68$<br>**p < 0.0001**	$\beta = +2.71$<br>$SE = 0.0619$<br>$F_{1,14421} = 1911.49$<br>**p < 0.0001**
Reward:Effort	$\beta = +0.224$<br>$SE = 0.0456$<br>$F_{1,14070} = 24.05$<br>**p < 0.0001**	$\beta = +0.235$<br>$SE = 0.0456$<br>$F_{1,14421} = 26.65$<br>**p < 0.0001**
Apcont	$\beta = -0.361$<br>$SE = 0.235$<br>$F_{1,14070} = 2.36$<br>$p = 0.12$	
Apcont:Effort	$\beta = +0.116$<br>$SE = 0.0454$<br>$F_{1,14070} = 6.56$<br>**p = 0.01**	
Reward:Apcont	$\beta = +0.136$<br>$SE = 0.0611$<br>$F_{1,14070} = 4.99$<br>**p = 0.026**	
Reward:Apcont:Effort	$\beta = +0.0869$<br>$SE = 0.0452$<br>$F_{1,14070} = 3.70$<br>$p = 0.059$	
Apcut		$\beta = -0.207$<br>$SE = 0.242$<br>$F_{1,14421} = 0.74$<br>$p = 0.39$
Apcut:Effort		$\beta = +0.24$<br>$SE = 0.0473$<br>$F_{1,14421} = 25.73$<br>**p< 0.0001**
Reward:Apcut		$\beta = +0.231$<br>$SE = 0.0641$<br>$F_{1,14421} = 12.93$<br>**p = 0.00032**
Reward:Apcut:Effort	1	$\beta = +0.108$<br>$SE = 0.0458$<br>$F_{1,14421} = 5.60$<br>**p = 0.018**
Nobs	14078	14429
adj − R^2	0.38	0.33
BIC	7341.42	7423.69

Drift diffusion model evaluation.

Assessing model convergence:

Our DDM chains appropriately converged as demonstrated in supplementary Figure 1. This was validated across six iterations of our model using the Gelman Ruben, or R-hat statistic, which assessed model convergence between and within MCMC chains. Typically R-hat values < 1.1 imply appropriate convergence. All computed R-hat values were < 1.001, and can be seen below in table 1.

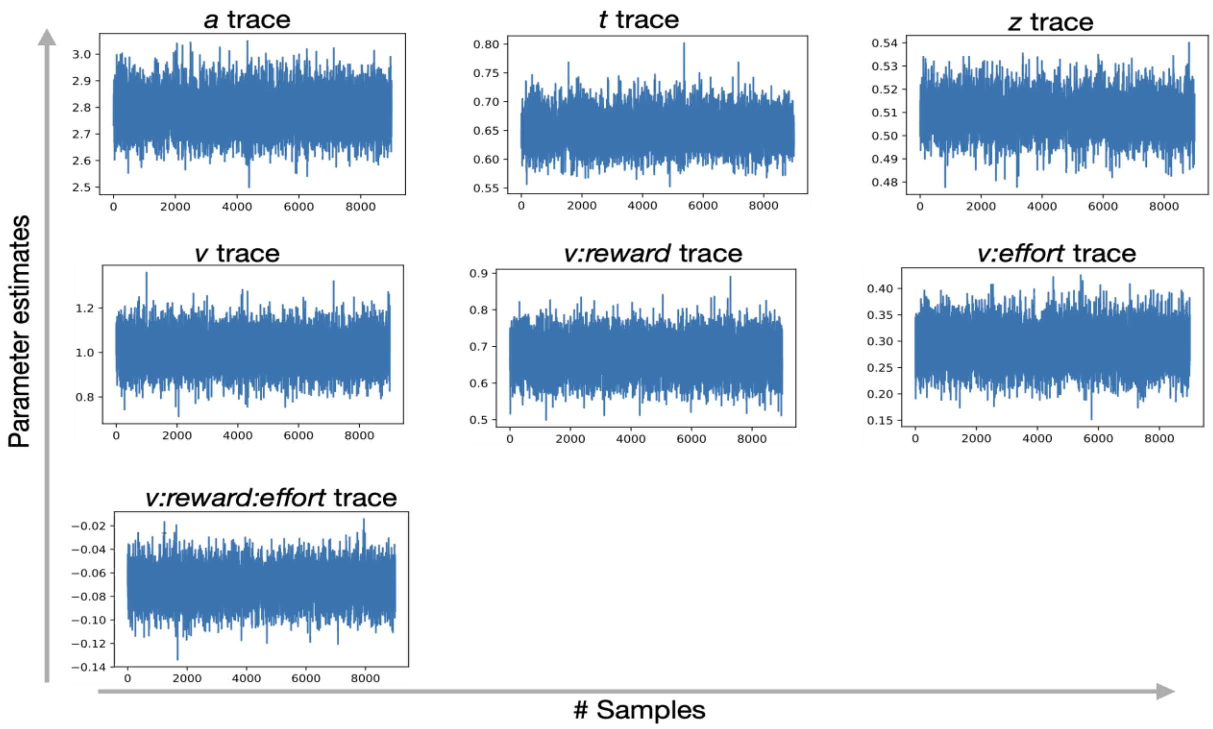

Supplementary Figure 1. Model convergence trace plots. All seven model parameters appropriately converged after 10,000 Markov chain Monte Carlo samples. The first 1,000 samples have been discarded as burn-in and are not shown here.

Parameter	R-hat
**Threshold (a)**	1.0000415570433712
**Bias (z)**	1.0007296051220003
**Non decision time (t)**	1.0000062930142812
**Baseline Drift rate (V)**	1.0000102057505023
**V:reward**	0.9999553245536233
**V:effort**	1.0000122137718024
**V:rew:Effort**	1.0000362395276252

Supplementary Table 1. Gelman Ruben statistic (R-hat) across six MCMC chains. Appropriate model convergence in our model as demonstrated by R-hat values < 1.1 across all seven model parameters.

Posterior Predictive checks:
To validate our model, simulated datasets were generated by sampling from our model's posterior distribution. Five hundred simulated reaction time (RT) datasets were generated using the '*hddm.utils.post_pred_gen'* function in the HDDM toolbox. These were subsequently compared to the true RT data using the '*hddm.utils.post_pred_stats'* function. Output summary statistics can be seen in table 2 below. These include comparisons between the proportion of offers accepted, as well as the RT distributions for both accepted and rejected offers between the true and simulated datasets. For each quantile of the RT distribution, the simulated data was probable to lie within the 95% interval of the true RT distributions and thus deemed "Credible" (see supplementary table 2 below). A plot of the posterior probability density of the simulated data can be seen in Supplementary figure 2 and closely approximates the original data.

STAT	OBSERVED DATA	SAMPLED DATA	STD	SEM	MSE	CREDIBLE
**ACCEPTED OFFERS (%)**	0.79	0.79	0.15	0.00	0.02	TRUE
**MEAN_RT ACCEPT**	1.63	1.80	0.77	0.03	0.62	TRUE
**STD_RT ACCEPT**	0.97	0.95	0.57	0.00	0.33	TRUE
**10Q_RT ACCEPT**	0.78	1.02	0.36	0.06	0.18	TRUE
**30Q_RT ACCEPT**	1.08	1.24	0.47	0.02	0.24	TRUE
**50Q_RT ACCEPT**	1.39	1.50	0.61	0.01	0.38	TRUE
**70Q_RT ACCEPT**	1.81	1.92	0.85	0.01	0.73	TRUE
**90Q_RT ACCEPT**	2.78	2.92	1.43	0.02	2.08	TRUE
**MEAN_RT REJECT**	-2.10	-2.21	0.90	0.01	0.82	TRUE
**STD_RT REJECT**	1.37	1.16	0.75	0.05	0.61	TRUE
**10Q_RT REJECT**	1.08	1.19	0.42	0.01	0.19	TRUE
**30Q_RT REJECT**	1.47	1.49	0.54	0.00	0.29	TRUE
**50Q_RT REJECT**	1.78	1.86	0.72	0.01	0.52	TRUE
**70Q_RT REJECT**	2.25	2.41	1.04	0.03	1.10	TRUE
**90Q_RT REJECT**	3.37	3.61	1.79	0.06	3.27	TRUE

Supplementary Table 2. Posterior predictive check summary. Sampled data from our model reproduced RT data for both accepted and rejected offers that were within the 95% credible interval of the observed RT data (credible column). This was consistent across all

Quantiles of the RT data. STD = Standard deviation; SEM = standard error of the mean; MSE = mean Squared error.

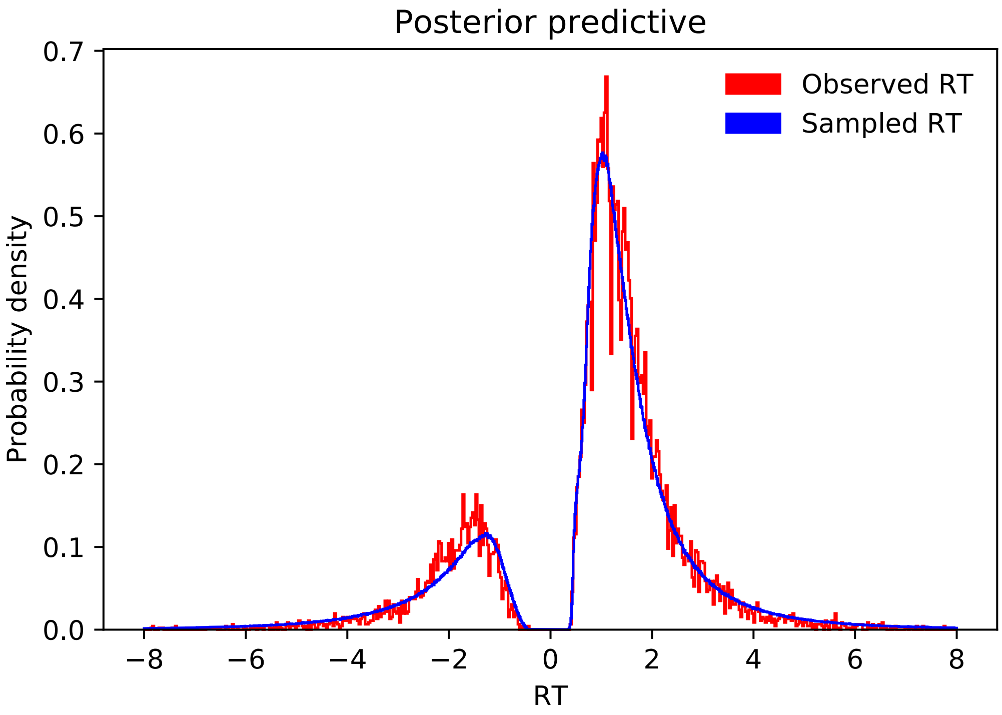

Supplementary figure 2. Posterior predictive plot for all patients**.** Probability density plot for all subjects' raw reaction time (RT) data (Red) in comparison to our model's predictions (blue). The two peaks represent the accepted trials' RT (positive x-axis values) and rejected trials' RT (negative values).

Does apathy drive increases in decision noise during decision making?

The drift rate is determined by the quality of information extracted from the stimulus (Ratcliff & McKoon, 2008). One possible hypothesis for the reduced drift rate observed in apathetic patients is that apathy is associated with an increase in decision noise. To test this hypothesis, we fit a variation of our original drift diffusion model, including per-subject parameter estimates of the inter-trial variability in the drift rate *sv* – a measure of noise. The model fit was marginally improved in comparison to our original model as measured by the DIC (28116 for new model vs 28119 for original model). All original associations between

apathy, depression and the DDM parameters were retained in this model. Specifically, apathy was still negatively associated with baseline drift rate while accounting for depression and age ($F(1,77)=4.28, p=0.042$). Crucially, there was no association between apathy and the inter-trial variability parameter *sv* ($F(1,77)=0.0089, p=0.92$). This suggests that increased decision noise is an unlikely mechanistic explanation for apathetic behaviour in this patient group.

Accounting for visible markers of SVD

A multiple regression was conducted including the FA values in the implicated TBSS tracts alongside two visible markers of SVD: Total lesion load and the number of lacunar infarcts. Only FA was significantly negatively associated with apathy.

	term	estimate	std.error	statistic	p.value	outcome
1	Intercept	-0.04	0.14	-0.26	0.80	Apathy
2	Lesion load	-0.07	0.12	-0.56	0.58	Apathy
3	Lacunes (number)	0.12	0.26	0.44	0.66	Apathy
4	FA	-0.36	0.13	-2.70	0.01	Apathy
5	Age	-0.22	0.13	-1.71	0.09	Apathy
6	Depression	0.41	0.12	3.36	0.00	Apathy

Supplementary table 3. Predictors of apathy in SVD. Multiple regression showing associations between apathy and several predictors including visible and non-visible markers of SVD. Significant associations highlighted with a red border.
